# Multipartite oil-flower/oil-bee mutualisms involving male-bee-pollinated orchids in tropical Asia

**DOI:** 10.1093/nsr/nwae072

**Published:** 2024-02-27

**Authors:** Meng Zhang, Li-Bing Jia, Susanne S Renner, Zhi-Xi Tian, Xiao-An Wang, Jiang-Yun Gao, Yi-Bo Luo, Antonio J C Aguiar, Huan-Li Xu, Shuang-Quan Huang

**Affiliations:** Institute of Evolution and Ecology, School of Life Sciences, Central China Normal University, China; Institute of Evolution and Ecology, School of Life Sciences, Central China Normal University, China; Department of Biology, Washington University, USA; Institute of Evolution and Ecology, School of Life Sciences, Central China Normal University, China; Institute of Evolution and Ecology, School of Life Sciences, Central China Normal University, China; State Key Laboratory for Conservation and Utilization of Bio-resources in Yunnan, Yunnan University, China; State Key Laboratory of Systematic and Evolutionary Botany, Institute of Botany, Chinese Academy of Sciences, China; Department of Zoology, University of Brasília, Brazil; Department of Entomology, College of Plant Protection, China Agricultural University, China; Institute of Evolution and Ecology, School of Life Sciences, Central China Normal University, China

Most animal-pollinated flowers offer nectar or pollen as a reward to their pollinators. A small fraction, however, have switched to other rewards, such as fatty oils offered in glandular hairs or just below the epidermis. This type of reward has evolved in some 150 genera in at least 11 families, including South American and African Orchidaceae [1,2]. Oil-offering flowers are exploited by a few hundred species of oil-collecting bees in temperate and tropical biomes on all continents except Antarctica [1,2]. Oil bees depend obligatorily on floral oil for cell lining and as larval food [3,4], which is why oil collecting usually is confined to female bees.

Differently from South America and Africa, oil-offering orchids have not been reported from tropical Australasia, despite the presence of oil-collecting bees in the genus *Ctenoplectra* that occur in Africa, Australia and Southeast Asia (China has four species) and that sweep up floral oil by moving the sternal hair brushes on their metasoma over the hair patches with repeated sidewise movements (Fig. 1, Fig. S1 and Video S1). In Asia, these bees were thought to depend exclusively on Cucurbitaceae, mostly species of *Momordica* and *Thladiantha* that bear glandular hairs on the inner corolla of the flowers [3,4] (Table S1). The only *Ctenoplectra* nests so far studied in Asia were built using a mixture of moderately fine soil and a substance, probably floral oil, that caused the soil particles to stick together [3].

**Figure 1.**
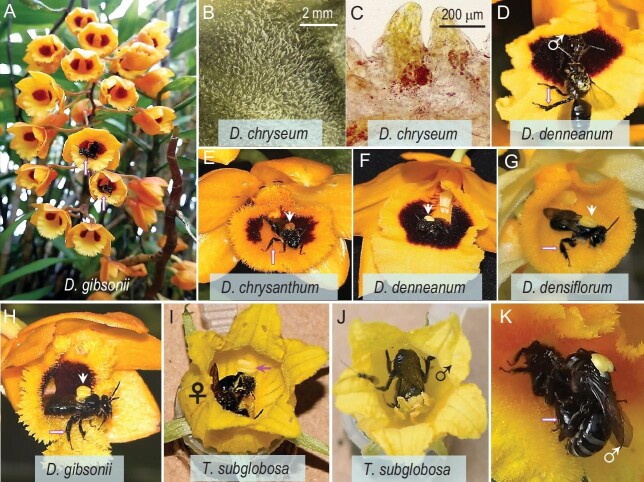
Habit, floral traits and oil bees on oil-offering *Dendrobium* and Cucurbitaceae species in Malipo County, southern Yunnan. (A) Inflorescences of *D. gibsonii*. (B) Glandular hairs covering the labellum in *D. chryseum*. (C) A hand section of the labellum, with the hairs stained with Sudan Ⅳ. (D) A male *Ctenoplectra cornuta* trying to enter a flower of *D. denneanum* that is occupied by another male bee (male symbol marked). (E–H) Male *C. cornuta* bees leaving the flowers of four *Dendrobium* species, each with pollinaria attached to their thorax (arrowheads). Note the characterized hind legs of males (arrow). (I) A female *C. cornuta* collecting nectar, oil and pollen from *Thladiantha subglobosa*, noting a pollinarium left on the corolla (arrow). (J) A male *C. cornuta* collecting nectar from *T. subglobosa*. (K) *Ctenoplectra* mating on *D. chryseum*, the male with a pollinarium on its back is delivering floral secretion (arrow) that he collected from *Dendrobium* flowers to the female. Photo credits: (A), Li-Bing Jia; (B), Shuan-Quan Huang and (C-K), Meng Zhang.

In this study, we describe the discovery of Asian oil-offering orchids in the genera *Dendrobium* and *Galeola* that surprisingly are exploited by males of *Ctenoplectra*, not females. As reported by Vogel [4], the females forage for nectar, pollen and oil on Cucurbitaceae, but males collecting floral oil from orchids had not been reported before. To document and quantify the extent of the newly discovered interactions, we carried out observations and experiments at the Centre for Gardening and Horticulture of the Xishuangbanna Tropical Botanical Garden (21.87°N, 101.32°E, 570 m alt.) from April to May in 2021 and 2023, and at an orchid breeding and conservation centre in Malipo County, Wenshan Autonomous Prefecture (23.18°N, 104.84°E, 1400 m alt.) from June to August in 2016, 2017, 2018, 2021, 2022 and 2023.

## MALE *CTENOPLECTRA* BEES COLLECT FLORAL OILS FROM *DENDROBIUM* AND *GALEOLA* ORCHIDS, WHEREAS FEMALES COLLECT OIL FROM CUCURBITACEAE

Of 39 species of *Dendrobium* and two species of *Galeola* that we tested for the presence of fatty oil in the minute hairs on their labella, 31 *Dendrobium* and both *Galeola* showed strong staining with Sudan III or Sudan IV as documented in the color photos in Table S2. During

hundreds of hours of observation, we observed pollination and/or pollinaria export or import in 12 of the *Dendrobium* and one of the *Galeola* species, always by *Ctenoplectra cornuta* Gribodo, 1892 (Tables S1 and S3, Fig. 1 and Video S2). Overall, we observed 265 (69 + 196) visits of male and 40 (4 + 36) visits of female *Ctenoplectra* bees to 11 *Dendrobium* species (Tables S1 and S3). Male and female *Ctenoplectra* can be distinguished by the females’ larger tibial spur (Fig. S2C vs. I). The close-set teeth of the spurs serve to squeeze the oil out of the sternal brushes. While the oil-collecting brushes on the metasoma (Fig. S2F and G vs. L and M) and legs of females (Fig. S2D and E vs. J and K) are larger and denser than the homologous structures in the males (Table S4), both sexes have these structures.

Based on body size (Table S4), male and female *Ctenoplectra* are both effective *Dendrobium* and *Galeola* pollinators, and this is also supported by pollinaria attached to both sexes, but males visited orchid flowers much more frequently than did females. All three *Ctenoplectra* bees observed on *Galeola lindleyana* were also males. As a result, the sex ratio of *Ctenoplectra* bees visiting *Dendrobium* (265/40 = 6.625) was significantly male-biased (Fisher's exact test, *P* = 2.21 E-18), while the sex ratio of bees visiting *Thladiantha* in our area, including a new species that we described [5], did not differ from 50:50 (106/100 = 1.06; Table S3C; *P* = 0.903).

When a male *C. cornuta* arrived at a *Dendrobium* or *Galeola* flower, it climbed into the centre of it and probed the nectar spur, often without success (next section). It would then turn around, its back would touch the anther cap and the pollinarium would be placed on the thorax when the bee withdrew from the flower (Fig. 1E–H and Video S2). Before leaving, male bees sat on the labellum, grooming themselves with their front legs and then using the metasoma tip and hind legs to collect the floral secretion from the labellum and smear it onto their bodies for many seconds. Males also used *Dendrobium* flowers as shelter during rainy days and would defend them against other males (Fig. 1D). *Ctenoplectra* were also observed copulating in flowers of both *Dendrobium chryseum* and *Thladiantha* (Fig. 1K). During copula, the male grasps the female scutellum with his mandibles and repeatedly brushes her metassomal fringes with his hind legs and her hind scopae with his mid-legs (Fig. 1K). When the male does this, it transfers a secretion onto the female, and indeed drops on the male's hairs can be seen in scanning electron microscope (SEM) images (Fig. S2K and N).

## THE ROLES OF NECTAR IN THE *CTENOPLECTRA*/ORCHID/CUCURBIT MULTIPARTITE INTERACTIONS

*Dendrobium* orchids usually have nectar spurs, although not all species regularly produce nectar (Table S1). The *Thladiantha* and *Momordica* flowers that we tested produced two to three times more nectar than did the orchid with the highest nectar reward, *D. chryseum* (5.18 ± 0.51 μL, *n* = 17 vs. 1.79 ± 0.07 μL, *n* = 50 and 36.0 ± 1.0% vs.

24.7 ± 0.4%: Table S5, which includes five *Dendrobium* species with nectar and five without). The five *Dendrobium* species lacking any nectar were still visited by *Ctenoplectra* males, proving that it was the oil-offering glandular hairs, not the nectar, that the males were seeking (Table S6). When *Dendrobium* inflorescences were set out on the ground (in water-filled vials) near *Thladiantha* flowers, visit frequencies (visits per flower per hour) to the *Dendrobium* flowers increased by hundreds of folds (Table S3), suggesting that the orchids benefitted from co-occurring with cucurbit flowers.

## THE EVOLUTIONARY TIME FRAME OF THE INTERACTION BETWEEN OIL-OFFERING ASIAN ORCHIDS AND *CTENOPLECTRA* BEES

Prior to this study, oil-offering flowers in Asia were only known from ∼30 species of *Thladiantha* and a few species of *Momordica, Siraitia* and *Indofevillea*, all pollinated by *Ctenoplectra* females [3,4]. Our discovery that *Dendrobium* and *Galeola* orchids also offer oil and depend predominantly on male *Ctenoplectra* for pollination raises the question of the time frame during which tropical Asian oil-offering orchids have inserted themselves into the Cucurbitaceae/*Ctenoplectra* pollination mutualism. The latter mutualism dates back to the Early Eocene, some 56 (67–44) mya [6], while the *Dendrobium* lineage minimally dates to the early Miocene, based on a *Dendrobium* leaf fossil from New Zealand (23–20 mya; [7]).

The minimally 15 orchid species for which *Ctenoplectra* pollination is newly documented here (Table S1) represent different lineages in a molecular clock-dated phylogeny that includes 319 of the estimated 1500 *Dendrobium* species occurring in the Indo-Asian, Australasian and Pacific regions ([8]: our Fig. S3). While the pollinator rewards in this large genus are insufficiently known and statistical support for the phylogeny is low, a conservative estimate is that oil-secreting hairs on the labellum may have evolved four times and are only a few million years old.

## BROADER SIGNIFICANCE

The discovery that males of oil-collecting bees use the setae on their legs to collect floral oil raises the question of what they do with the oil, as males do not provision larvae nor engage in nest building. The most plausible explanation is that the oil plays a role during copulation, as the males grasp the females and repeatedly brush the females’ metassomal fringes with their hind legs and the females’ hind scopae with their mid-legs (Fig. 1K and Fig. S2). Males of Neotropical oil bees (belonging to *Tetrapedia* and *Paratetrapedia*) sometimes collect floral oil from Malpighiaceae or Araceae, but it is unknown for which purpose [9,10].

The discovery that many *Dendrobium* and some *Galeola* species offer oil and are pollinated by male *C. cornuta*, the females of which depend on Cucurbitaceae oil and pollen, highlights the challenge of maintaining or restoring natural orchid pollination mutualisms. Further studies of the multidimensional pollination mutualism involving male and female *Ctenoplectra* are needed to unveil the floral cues that attract the males to orchid floral oil but the females to cucurbit oil.

## Supplementary Material

nwae072_Supplemental_Files
