# Supplementary material for: Multipartite oil-flower/oil-bee mutualisms involving male-bee-pollinated orchids in tropical Asia
Source: Natl Sci Rev. 2024 Feb 27;11(5):nwae072. doi: 10.1093/nsr/nwae072 (PMC11127695; doi:10.1093/nsr/nwae072)
Supplement: nwae072_Supplemental_Files [file nwae072_supplemental_files.zip › Table_S2_Dendrobium_and_Galeola_trichomes_Sudan_stained_2024Jan.pdf]

Table S2. Fresh, intact flowers of *Dendrobium* and *Galeola* stained with saturated ethanolic solutions of Sudan III or IV to detect the presence of accumulated lipids. For each species, a cross section of a labellum shows the glandular hairs before staining and after staining under a microscope or stereoscope. Where lipids could not be detected, this is indicated by 'Not stained'. Voucher specimens are listed below the photos of each species and have been deposited in Herbarium of Central China Normal University (CCNU), see Table S1.

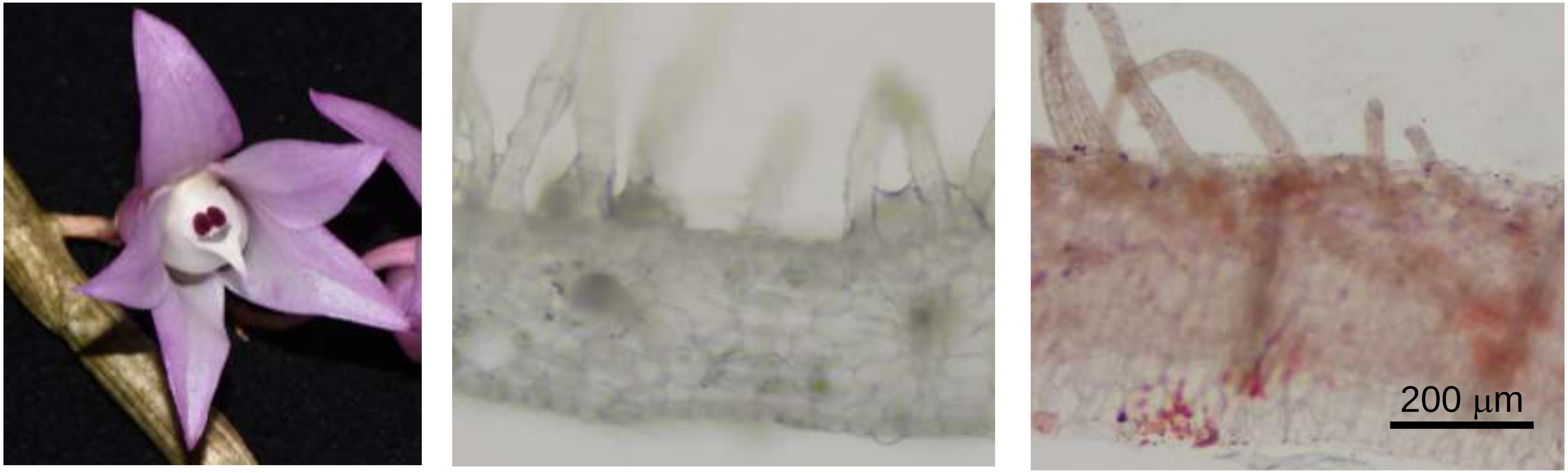

Dendrobium aduncum; Specimen no. M. Zhang 220625

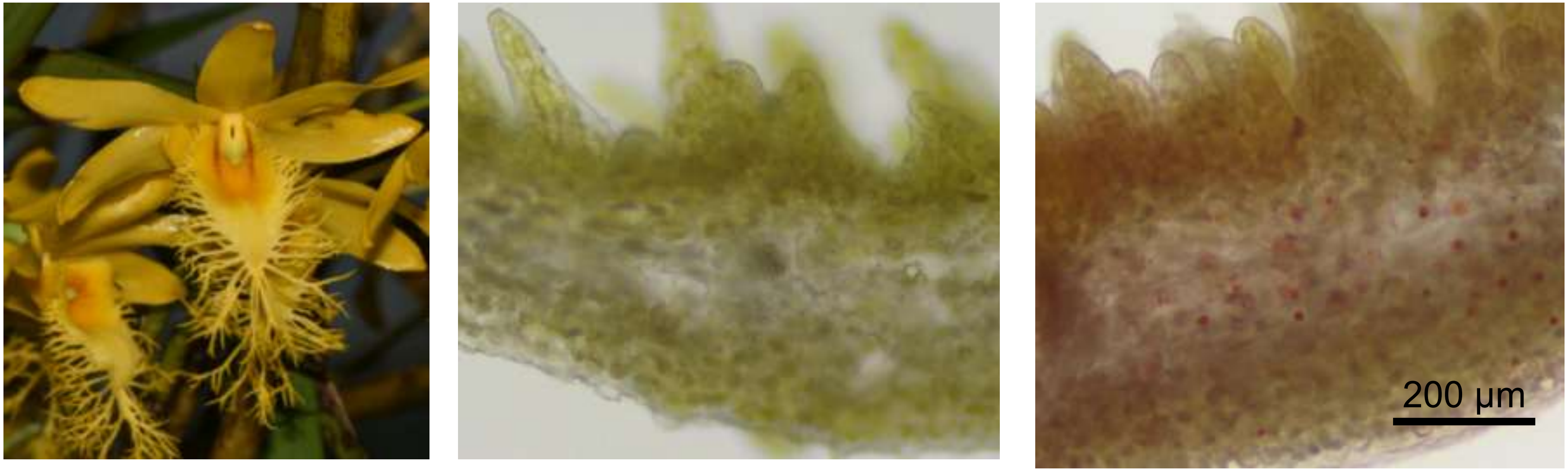

Dendrobium brymerianum; M. Zhang 220523

Before staining | Stained (Sudan IV)

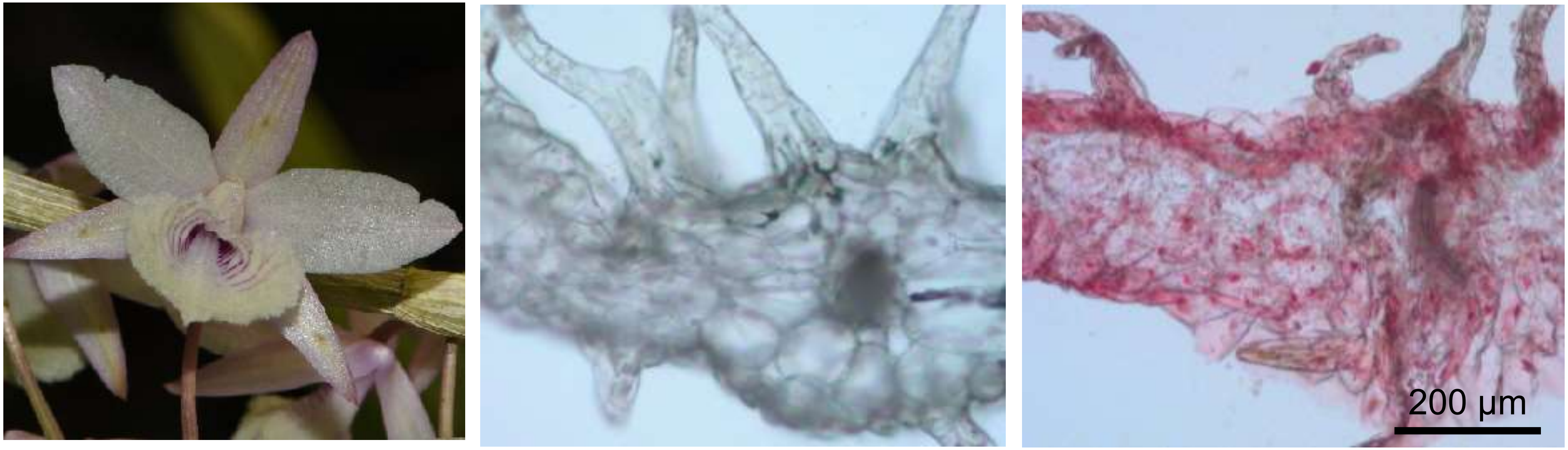

Dendrobium aphyllum; M. Zhang 220404

Not stained (Sudan IV)

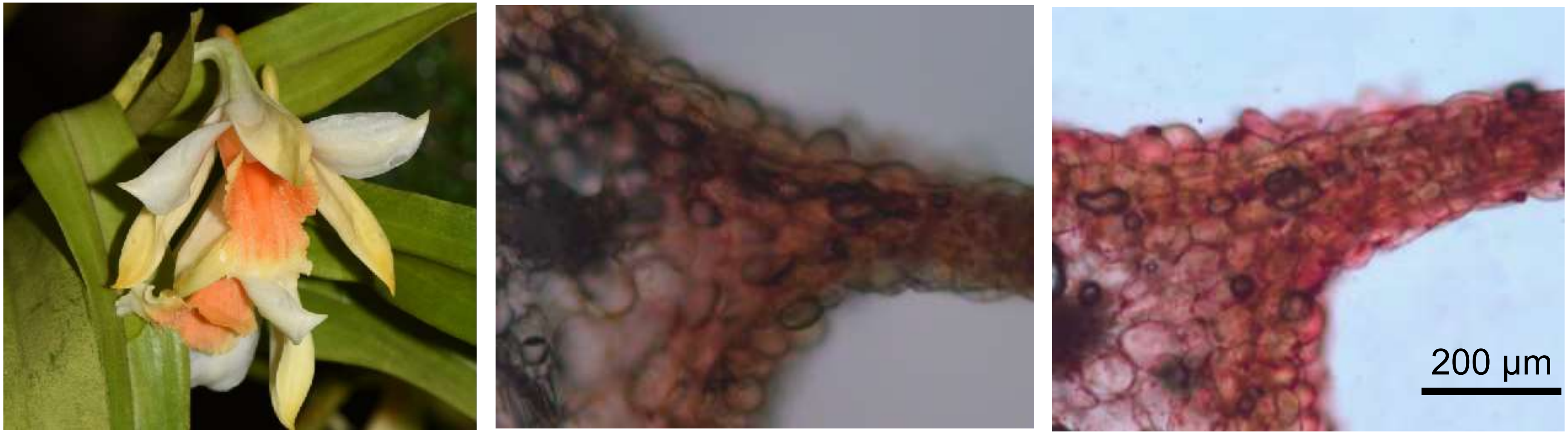

Dendrobium cariniferum; M. Zhang 220406

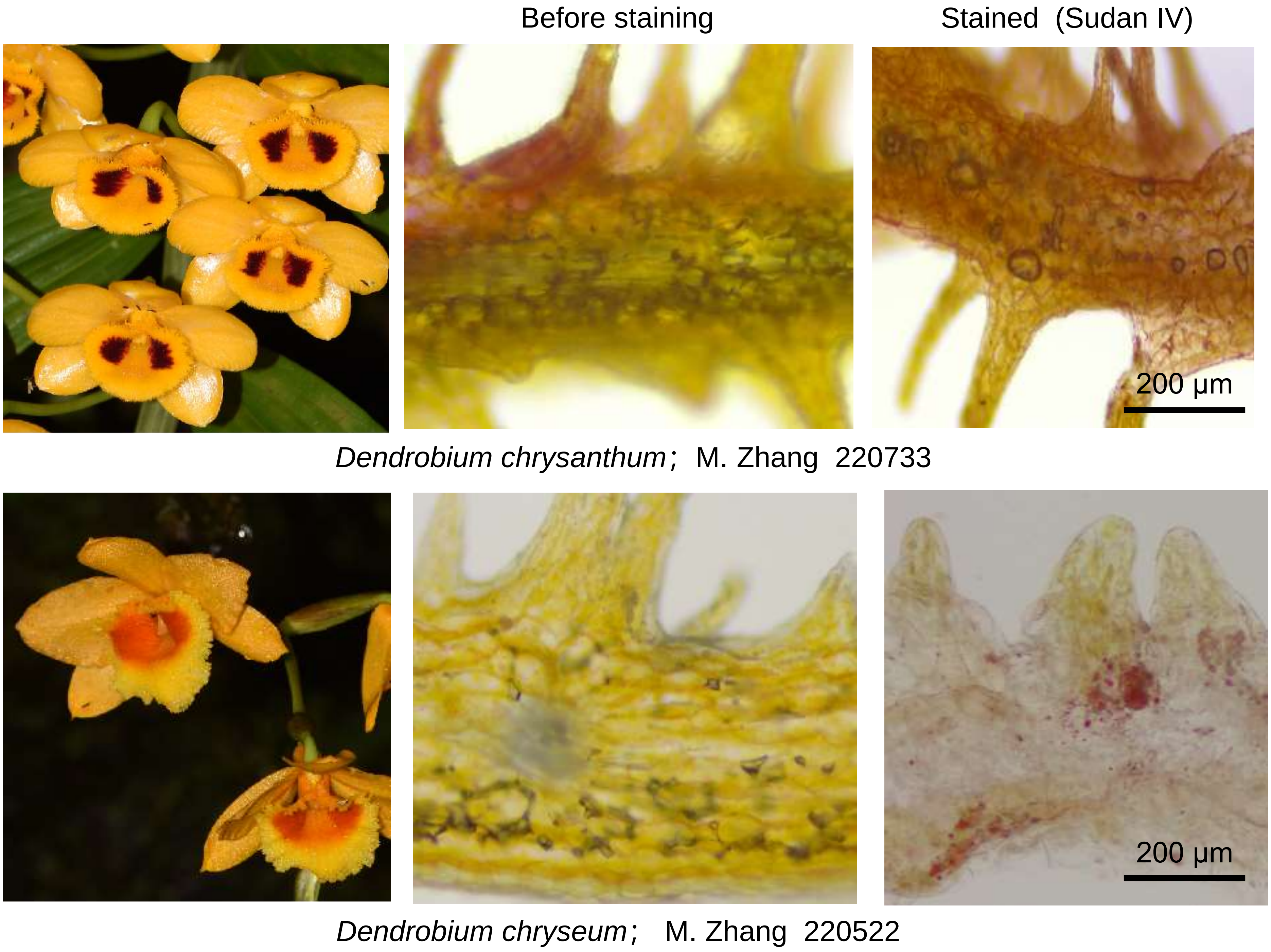
Before staining
Stained (Sudan IV)
200 µm
Dendrobium chrysanthum; M. Zhang 220733
200 µm
Dendrobium chryseum; M. Zhang 220522

Before staining Stained (Sudan IV)

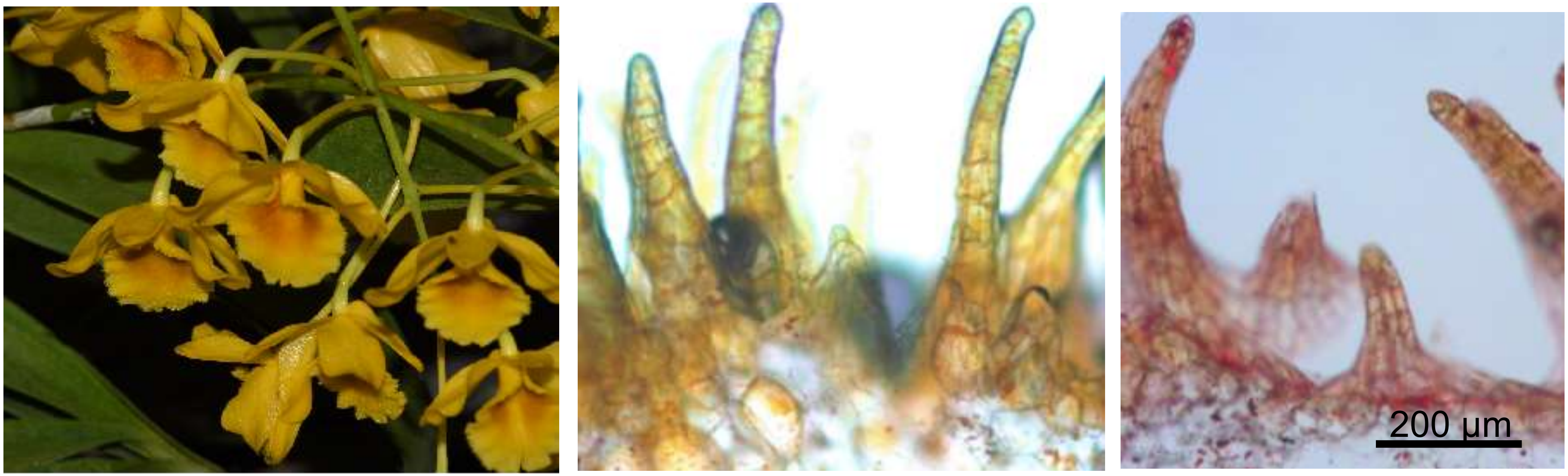

Dendrobium chrysotoxum; M. Zhang 220402

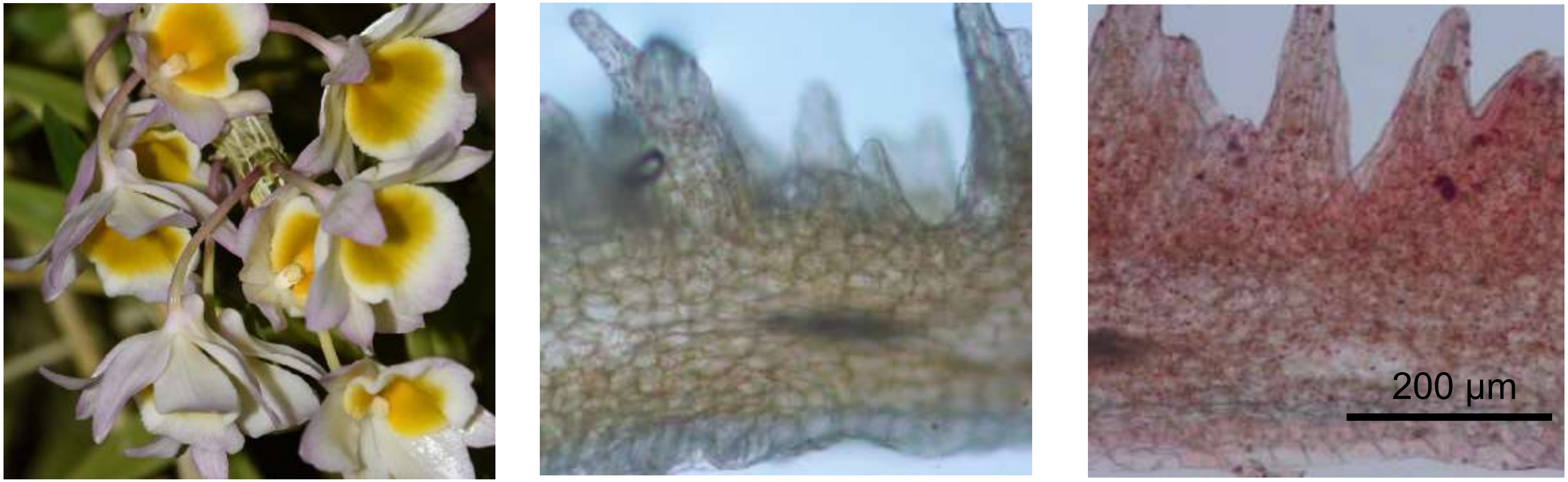

Dendrobium crepidatum; M. Zhang 220407

Before staining Not Stained (Sudan IV)

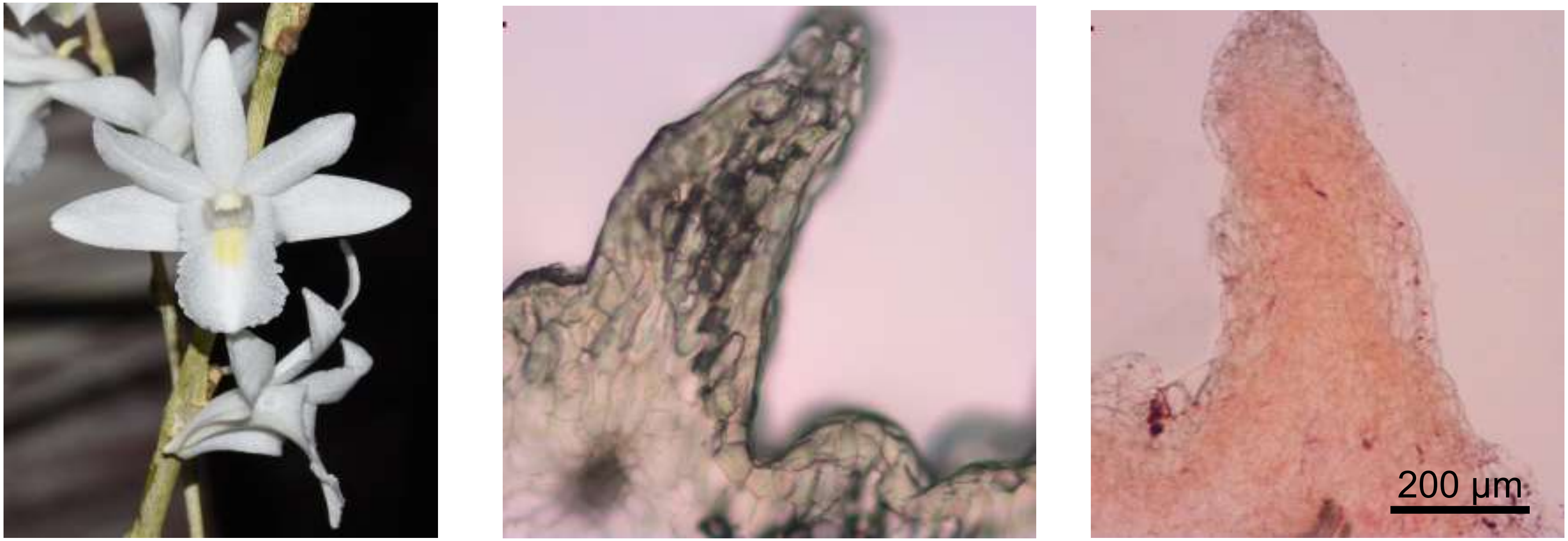

Dendrobium crumenatum; M. Zhang 220416

Stained (Sudan IV)

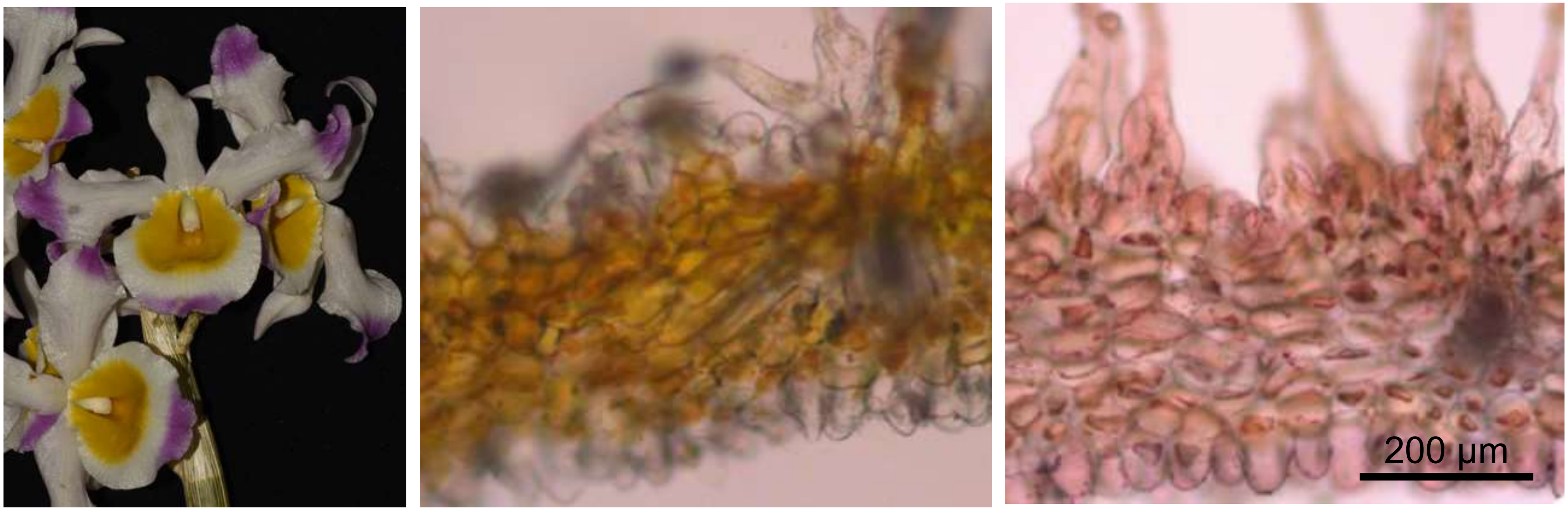

Dendrobium crystallinum; M. Zhang 220417

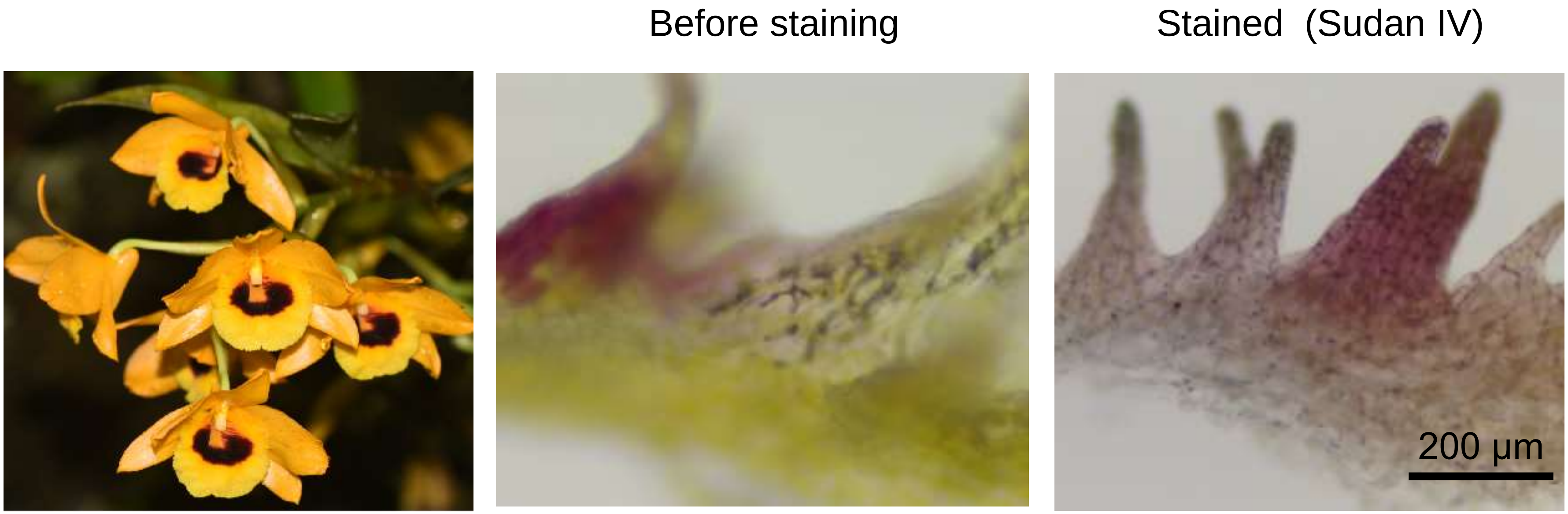

Dendrobium denneanum; M. Zhang 220524

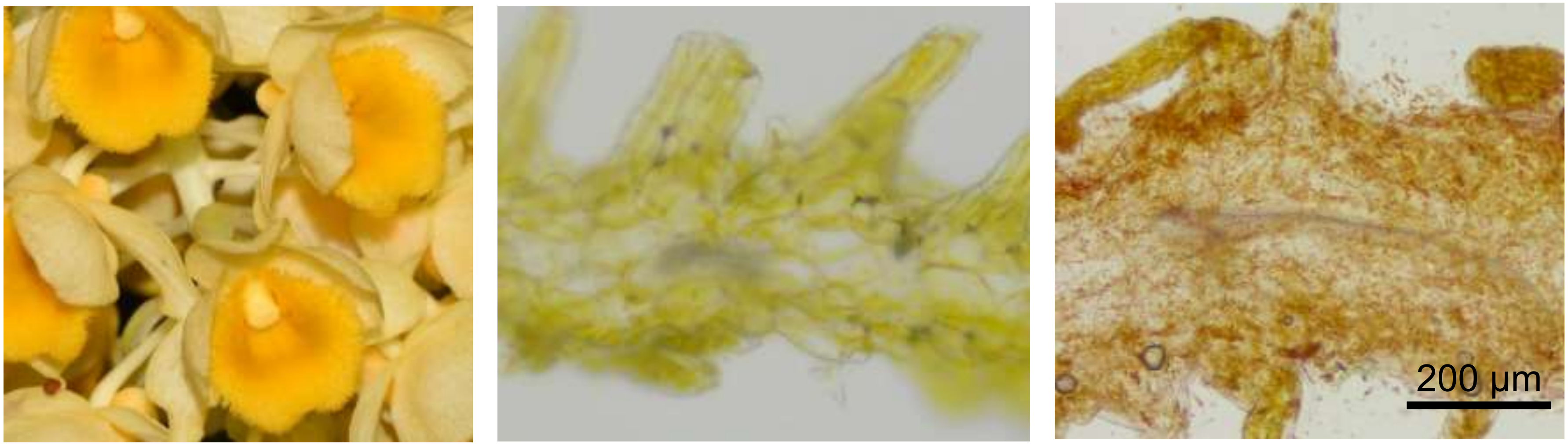

Dendrobium densiflorum; M. Zhang 220521

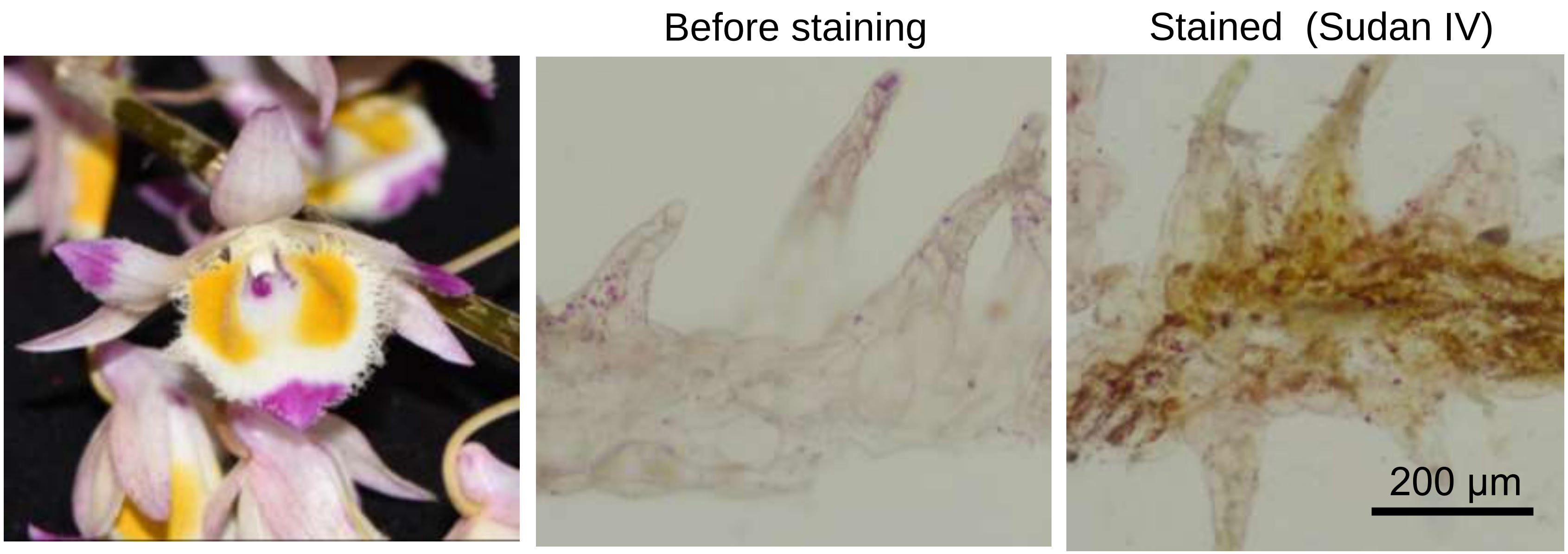

Dendrobium devonianum; M. Zhang 220627

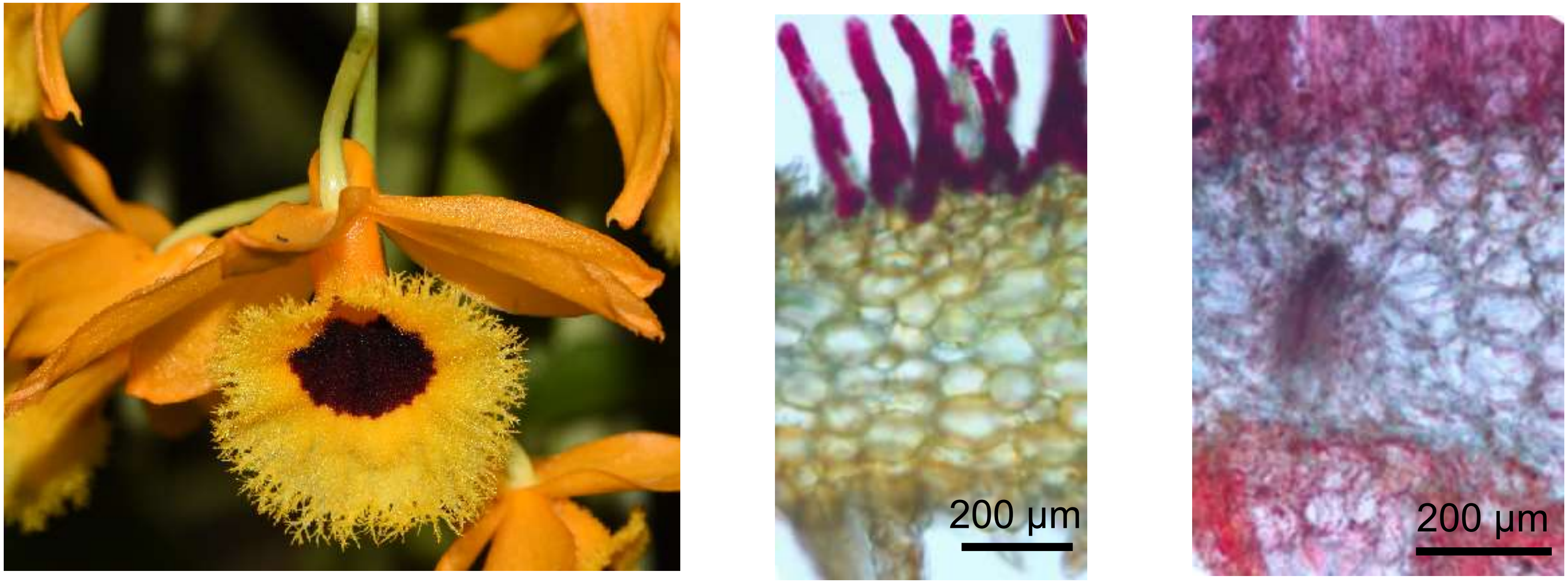

Dendrobium fimbriatum; M. Zhang 220403

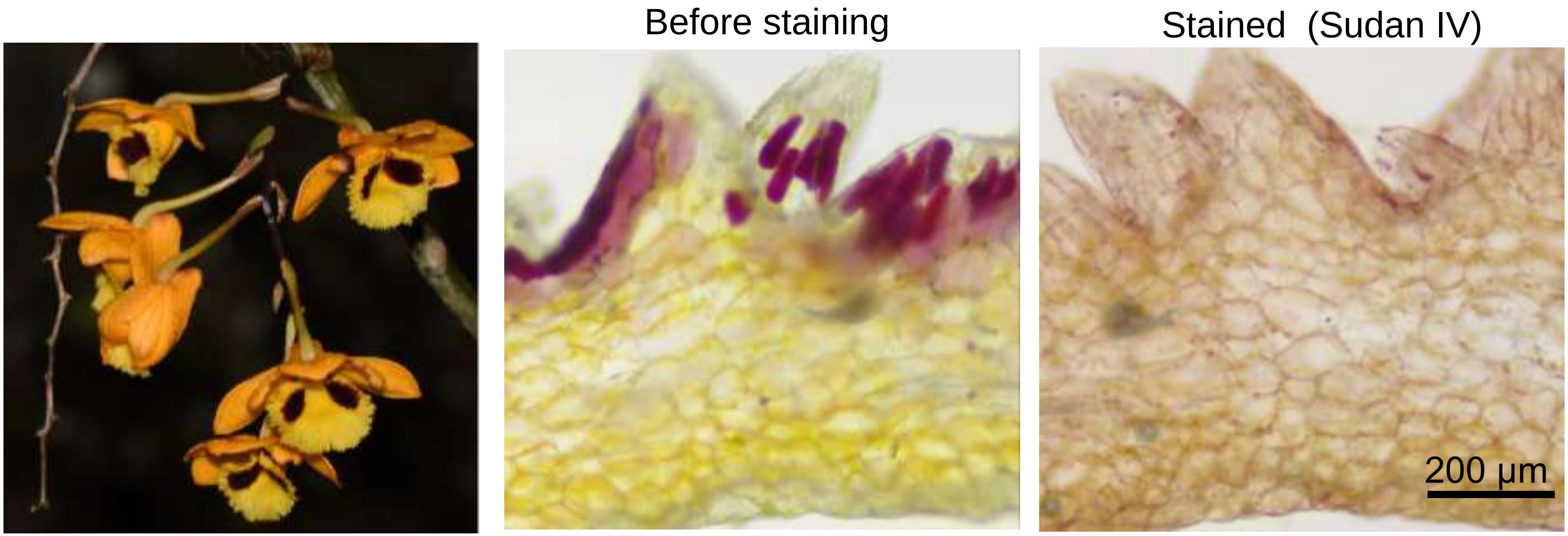

Dendrobium gibsonii; M. Zhang 220630

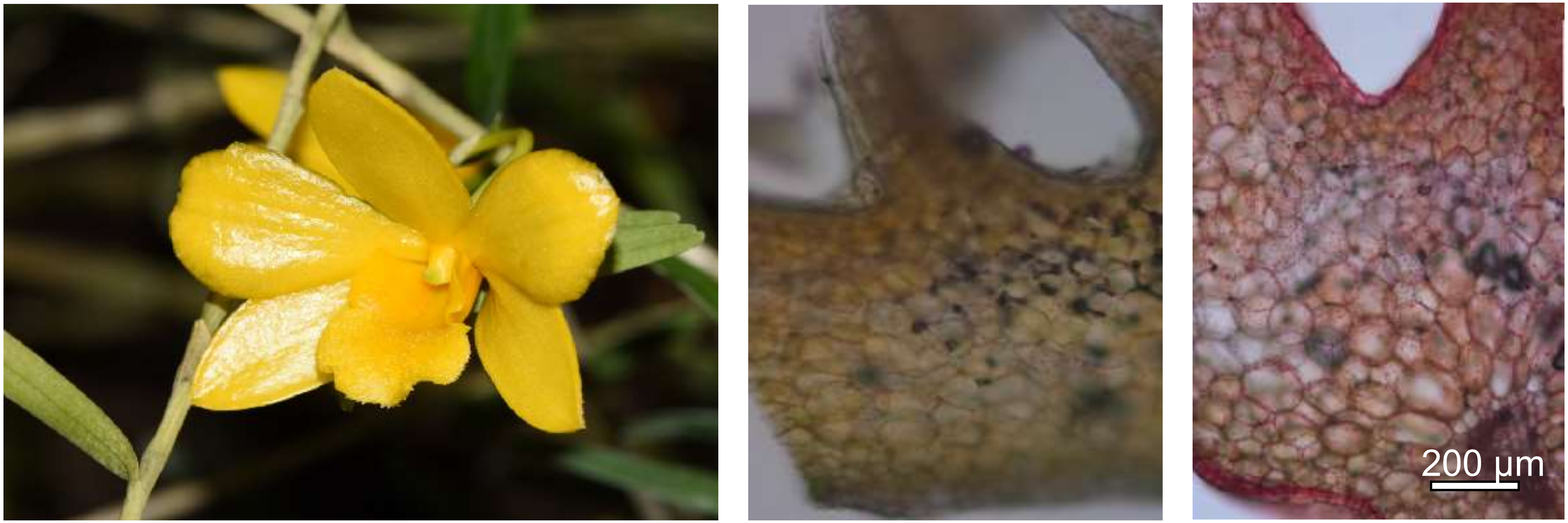

Dendrobium hancockii; M. Zhang 220414

Before staining Stained (Sudan IV)

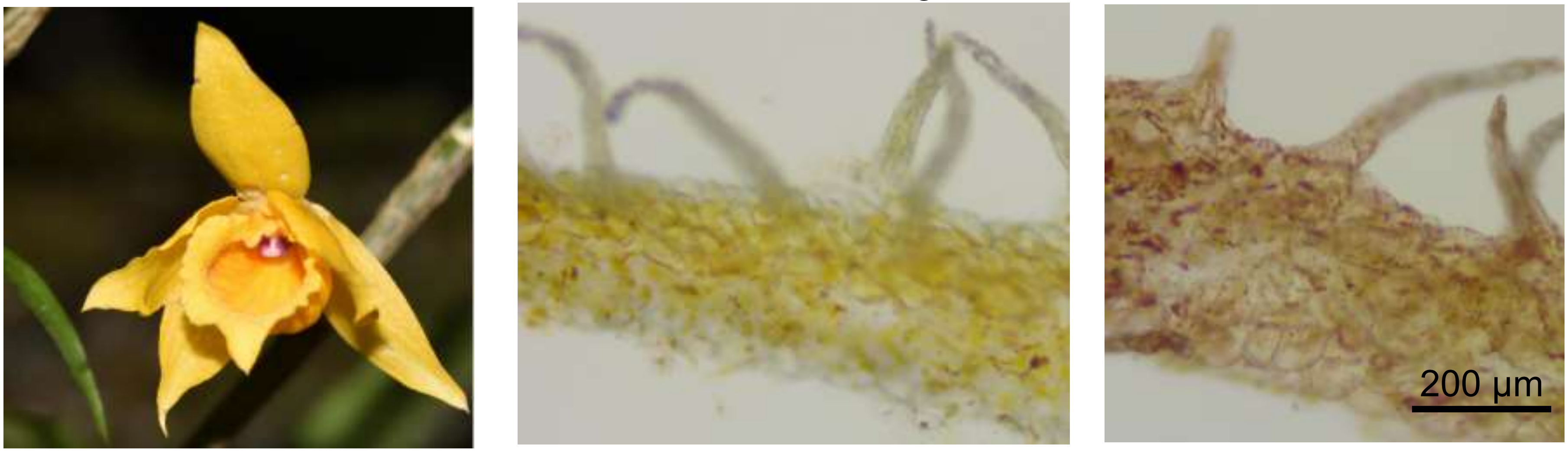

Dendrobium henryi; M. Zhang 220629

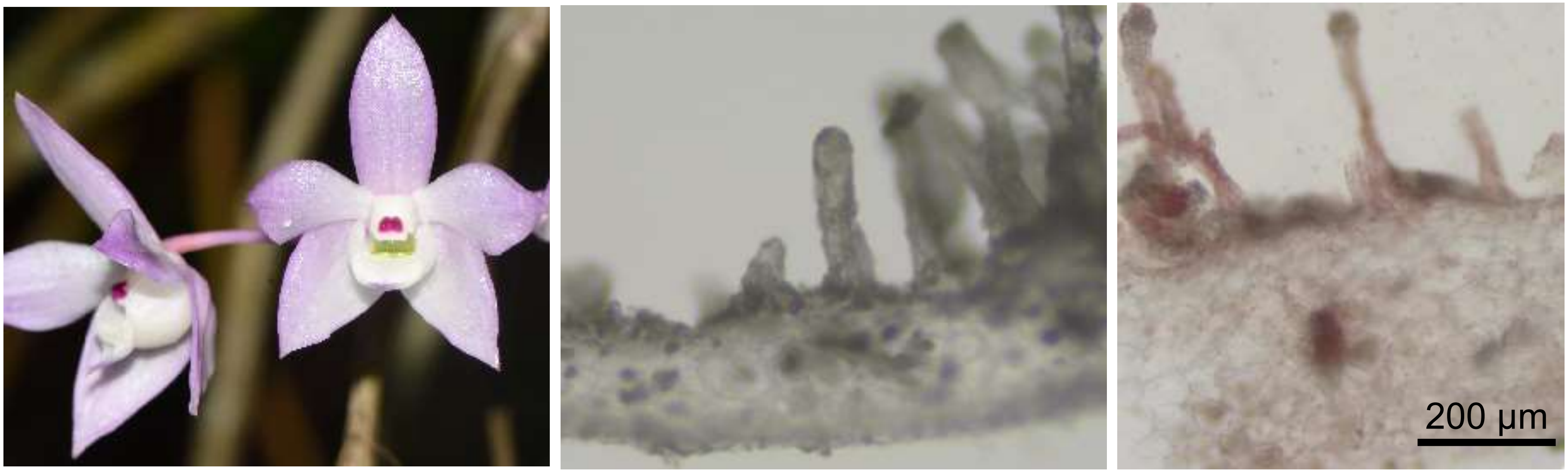

Dendrobium hercoglossum; M. Zhang 220628

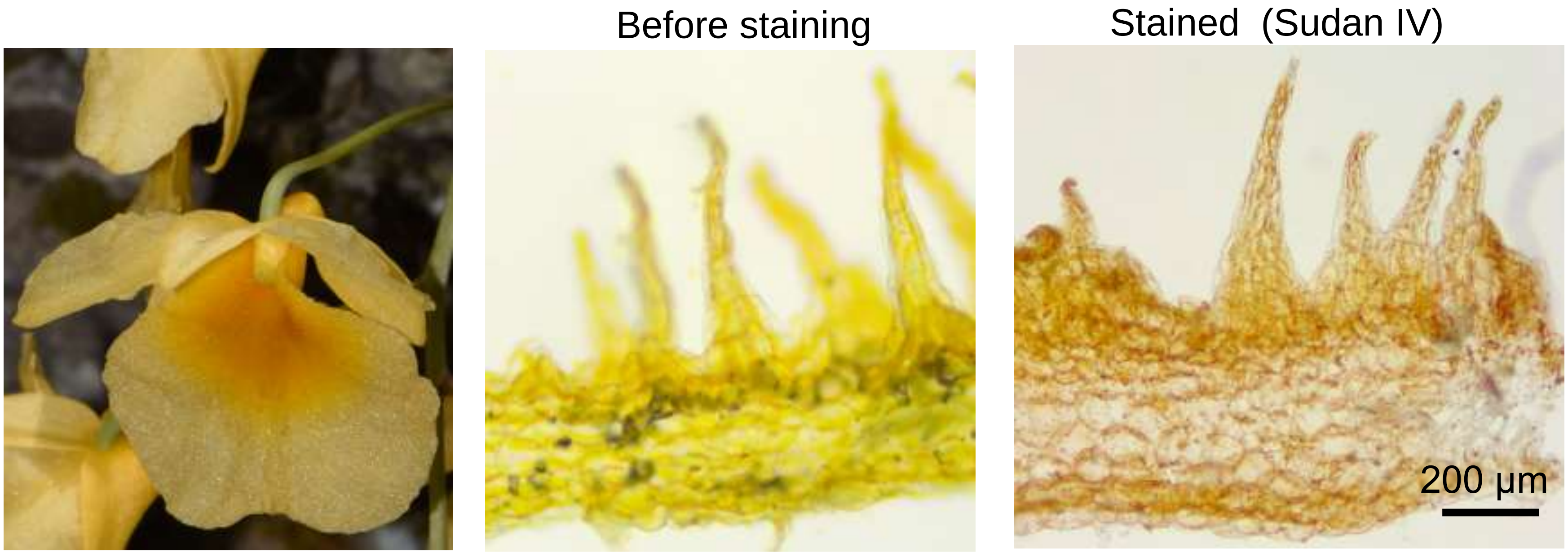

Dendrobium lindleyi; M. Zhang 220834

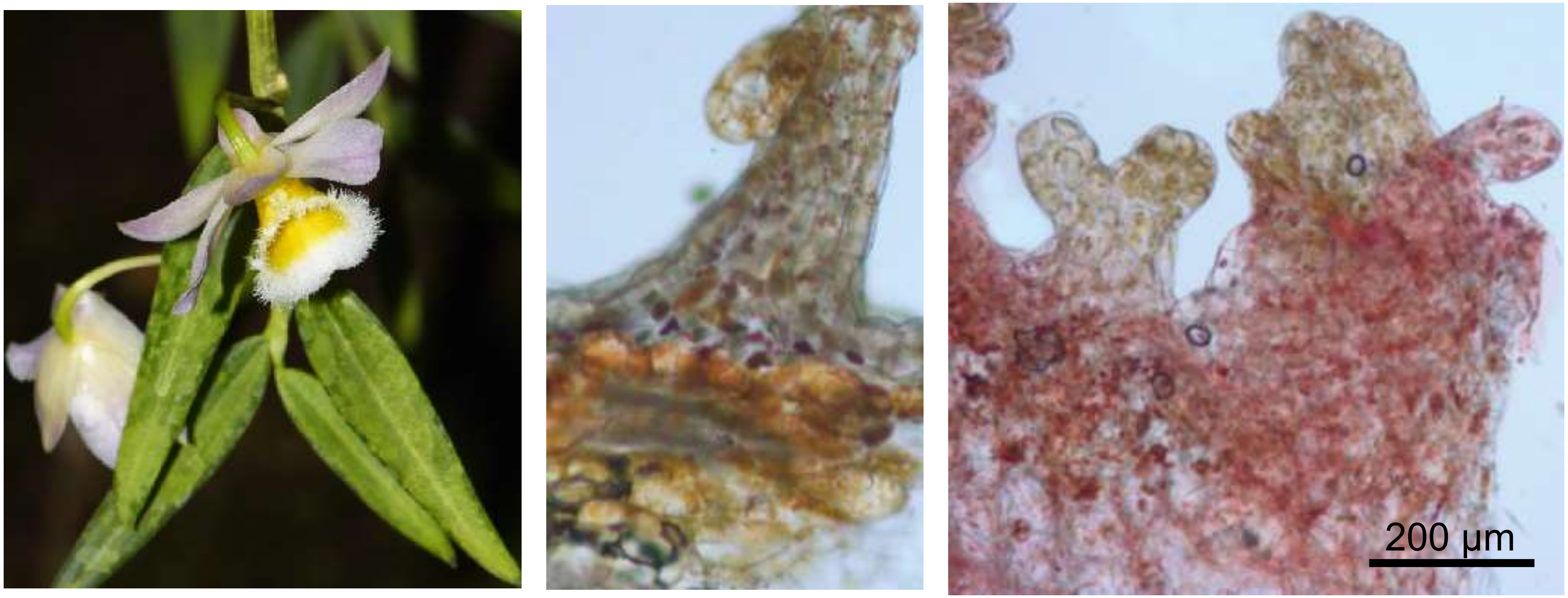

Dendrobium loddigesii; M. Zhang 220405

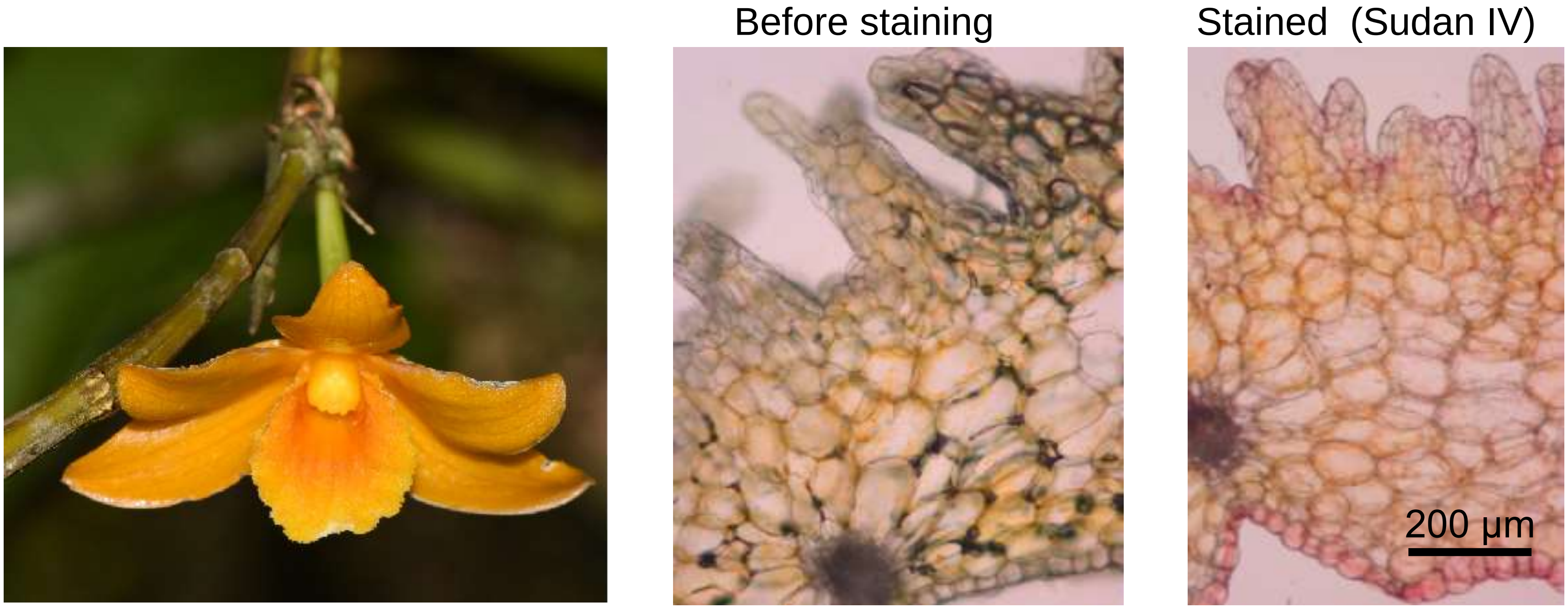

Dendrobium lohohense; M. Zhang 220520

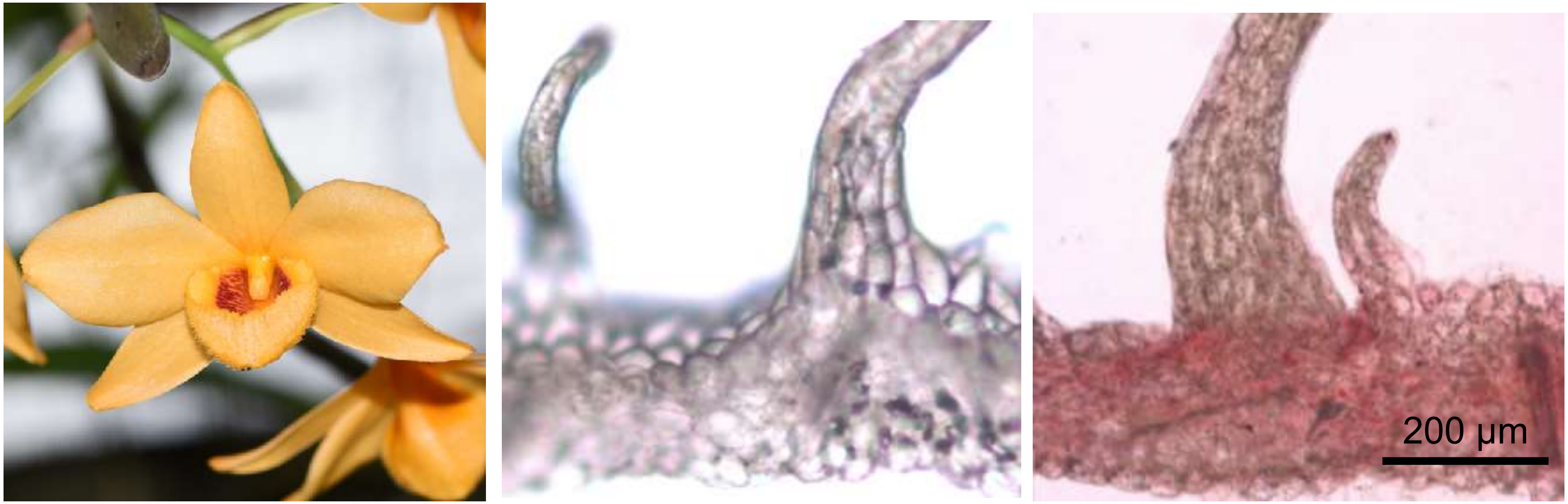

Dendrobium moschatum; M. Zhang 220418

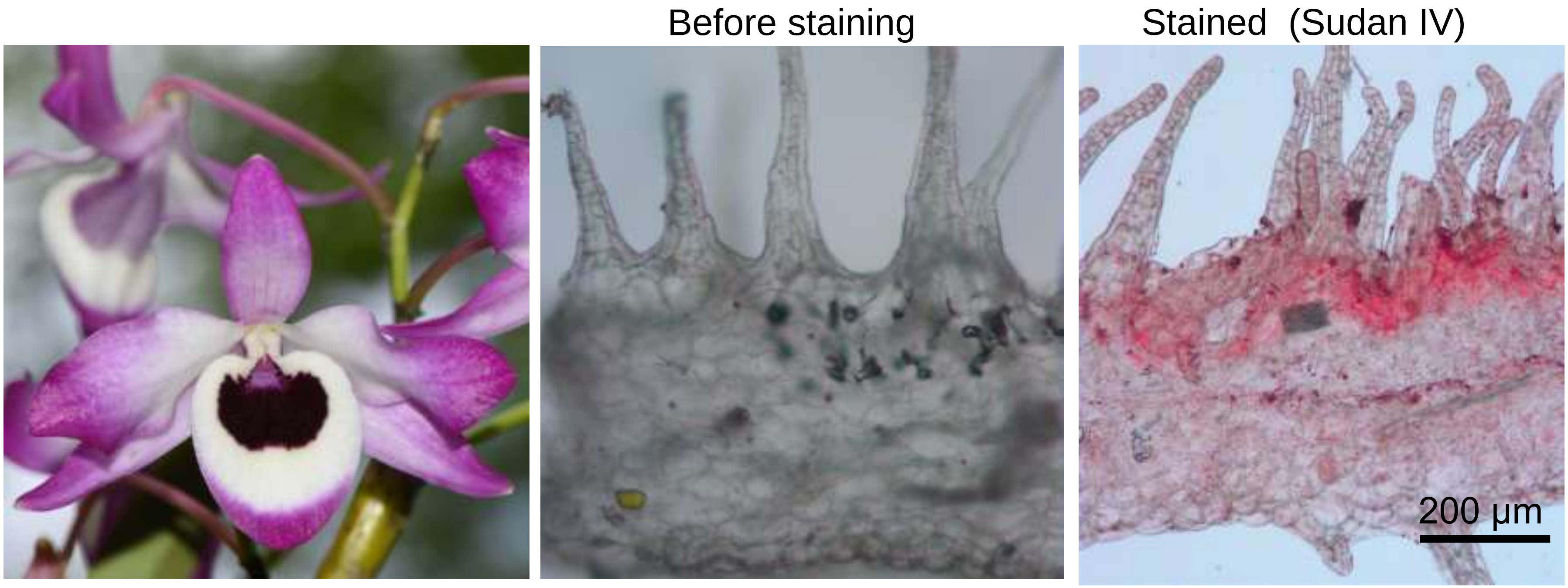

Dendrobium nobile; M. Zhang 220410

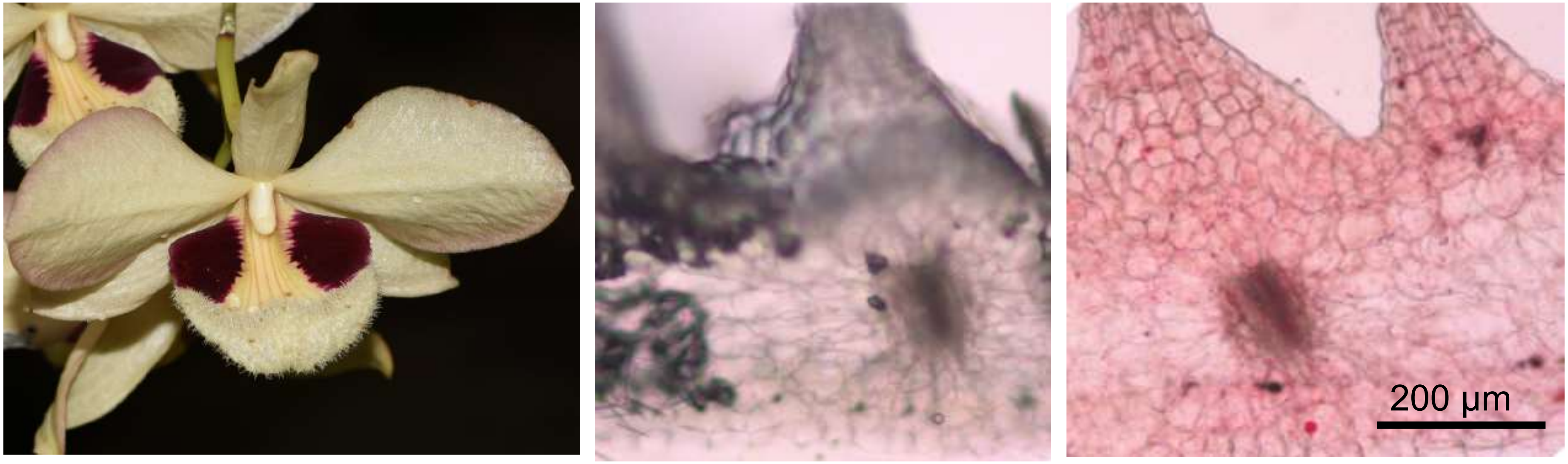

Dendrobium pulchellum; M. Zhang 220413

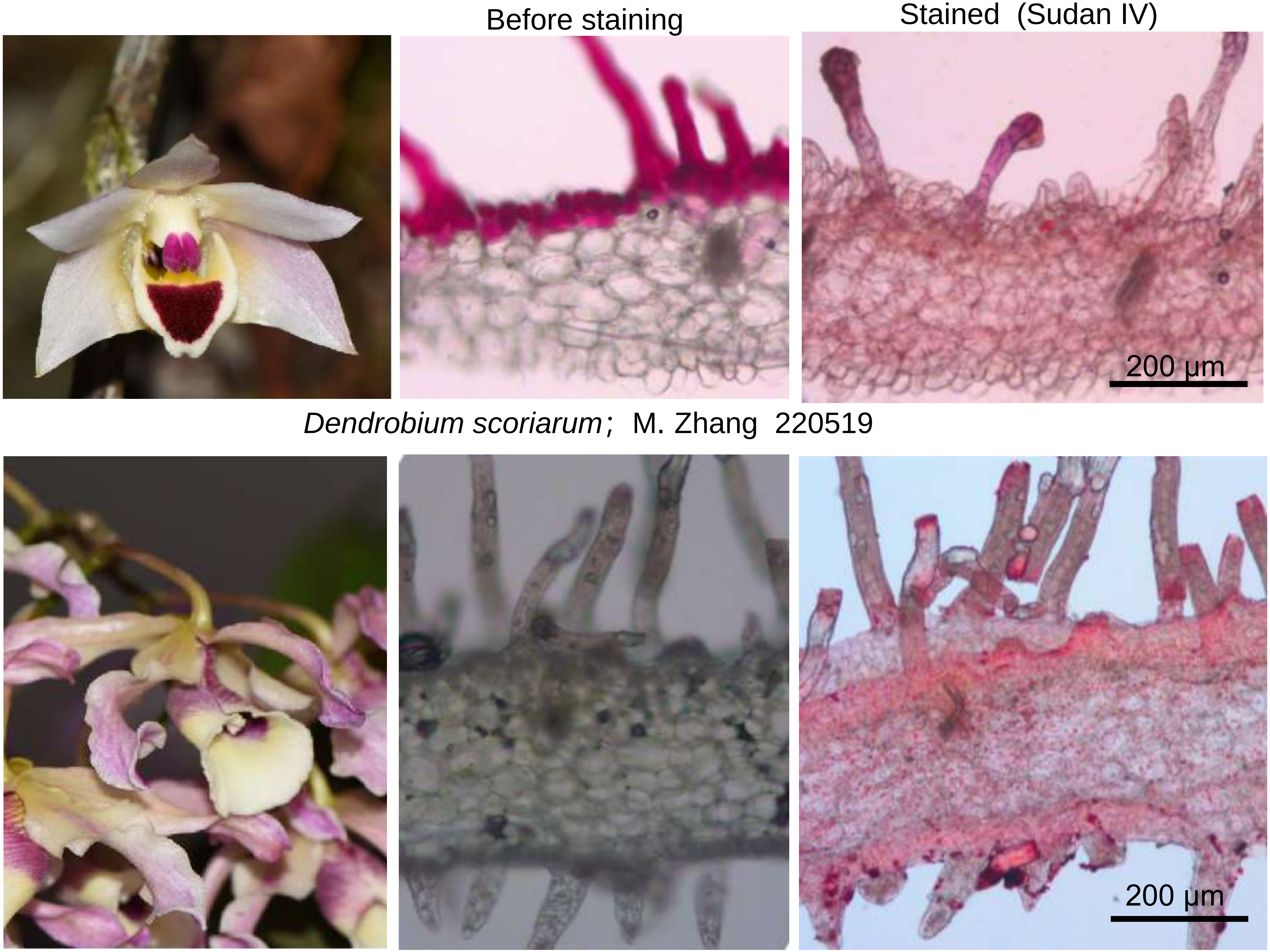

Dendrobium signatum; M. Zhang 220408

Before staining Stained (Sudan IV)

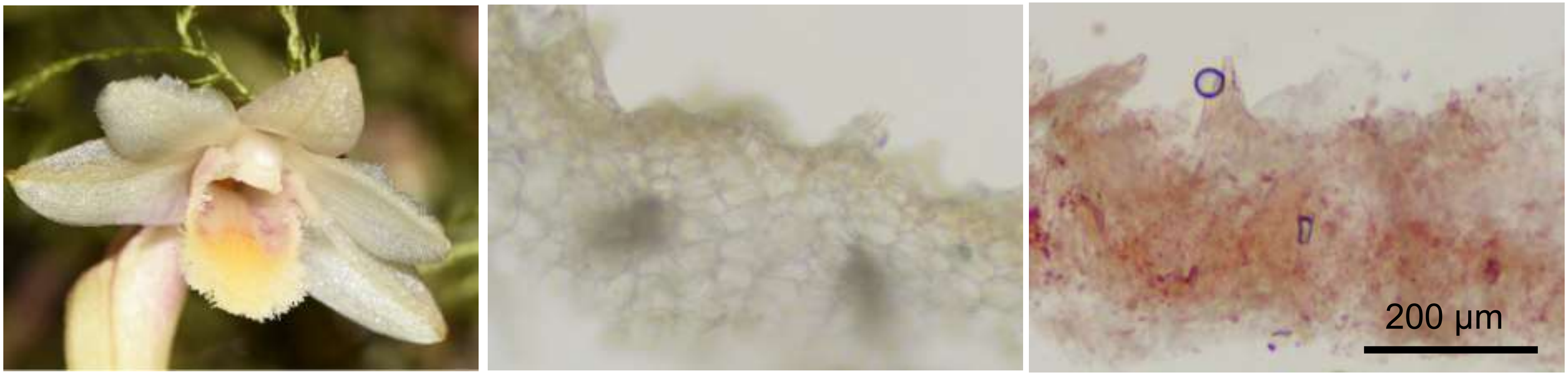

Dendrobium stuposum; M. Zhang 220731

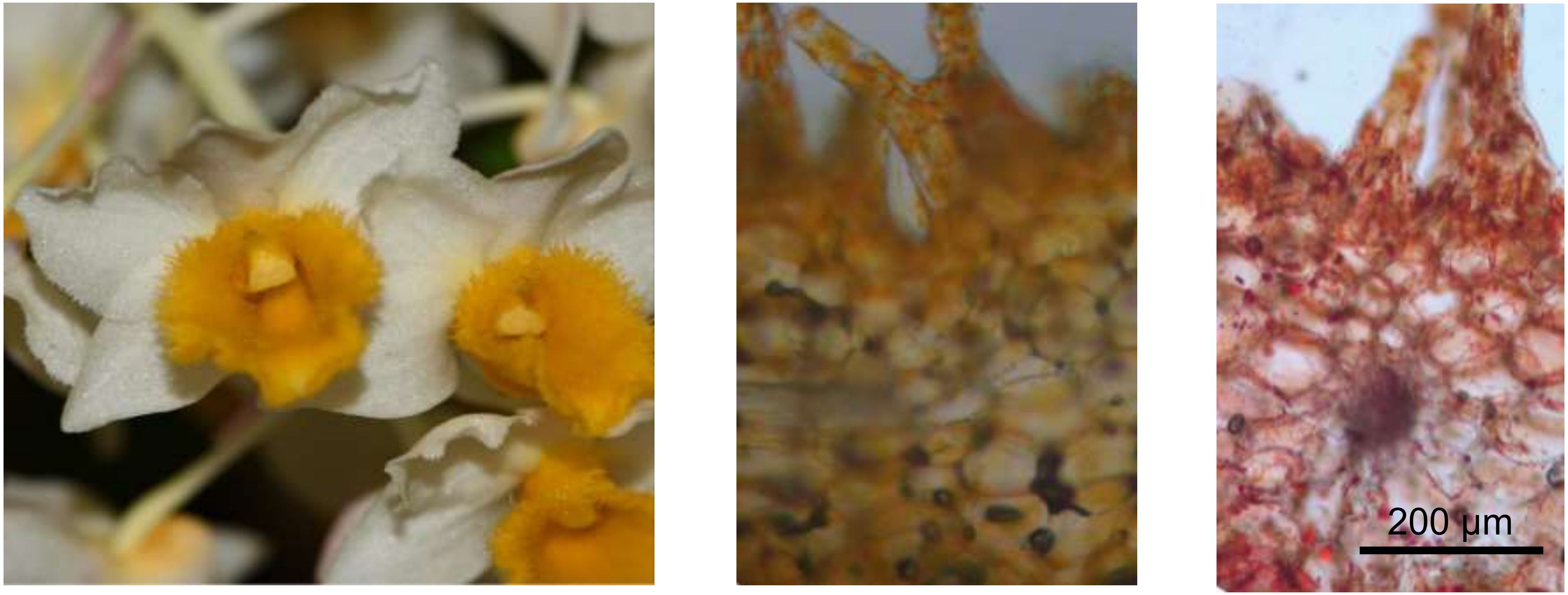

Dendrobium thyrsiflorum; M. Zhang 220401

Before staining   Stained (Sudan IV)

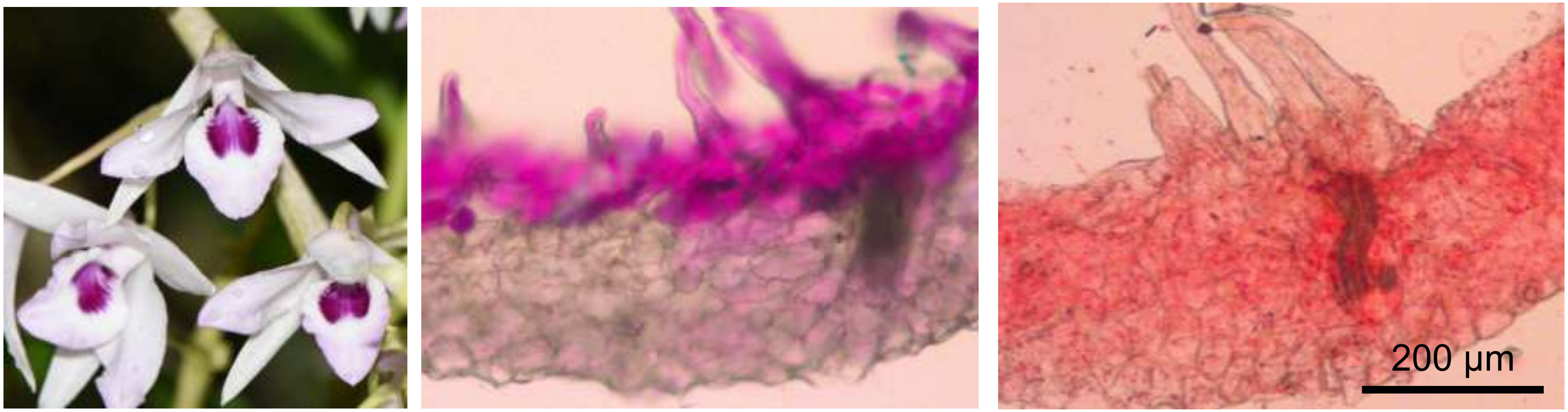

Dendrobium transparens; M. Zhang 220415

Not Stained (Sudan IV)

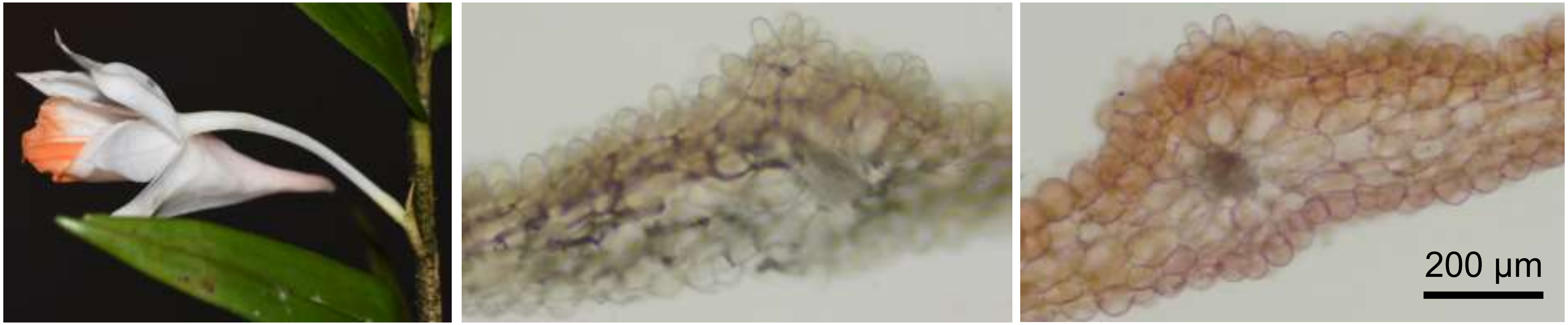

Dendrobium longicornu; M. Zhang 220732

Before staining Not Stained (Sudan IV)

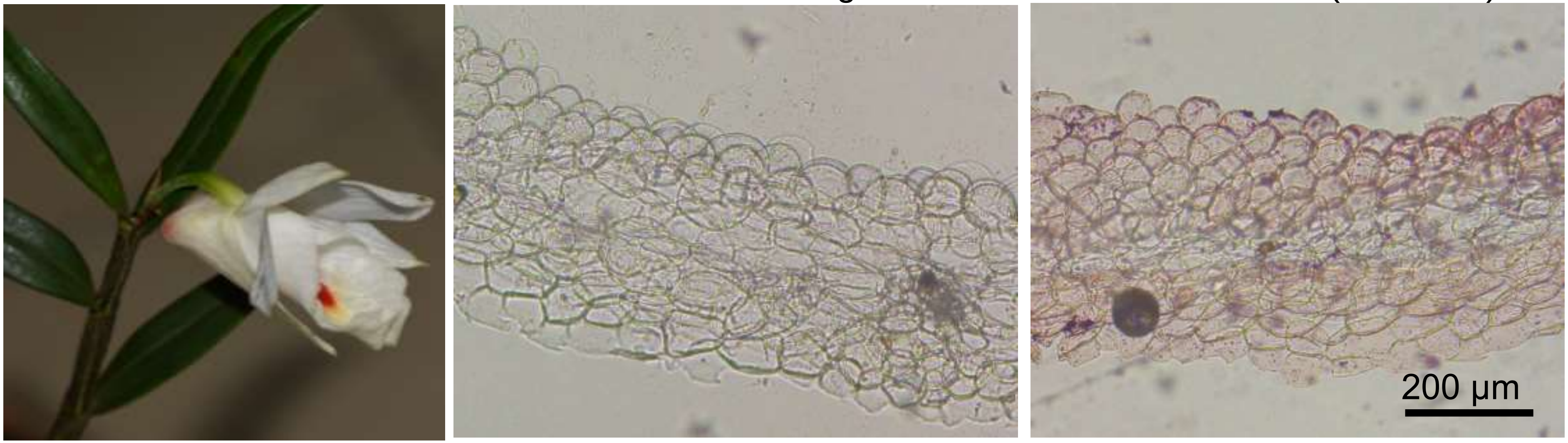

Dendrobium sinense; M. X. Ren 231031

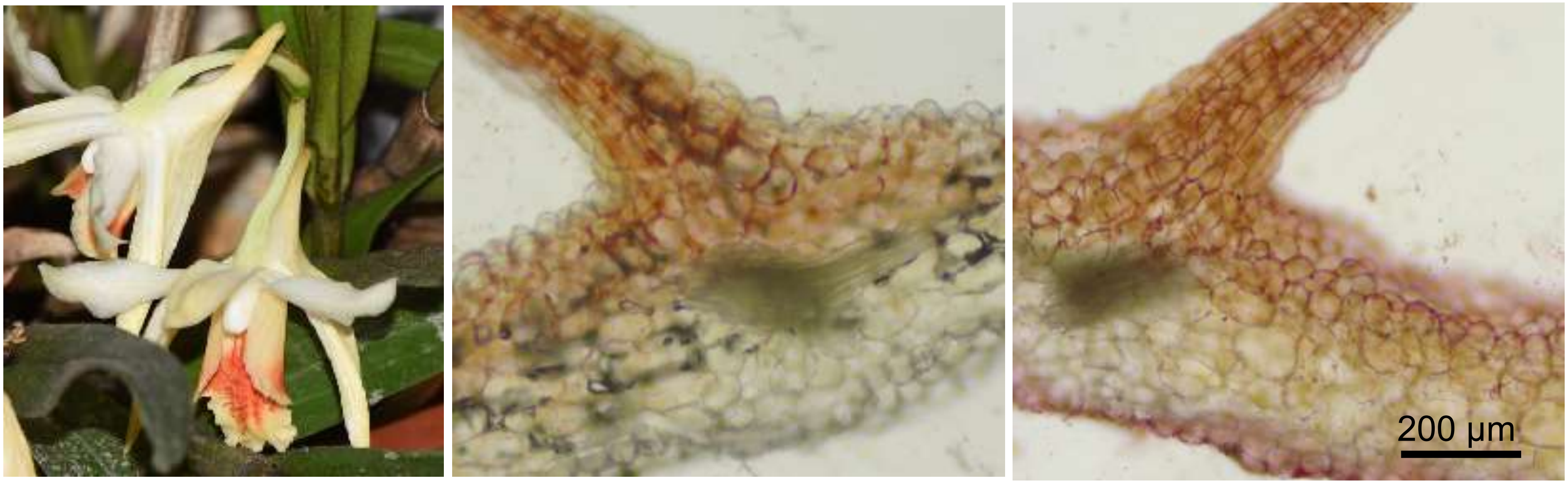

Dendrobium williamsonii; M. Zhang 220626

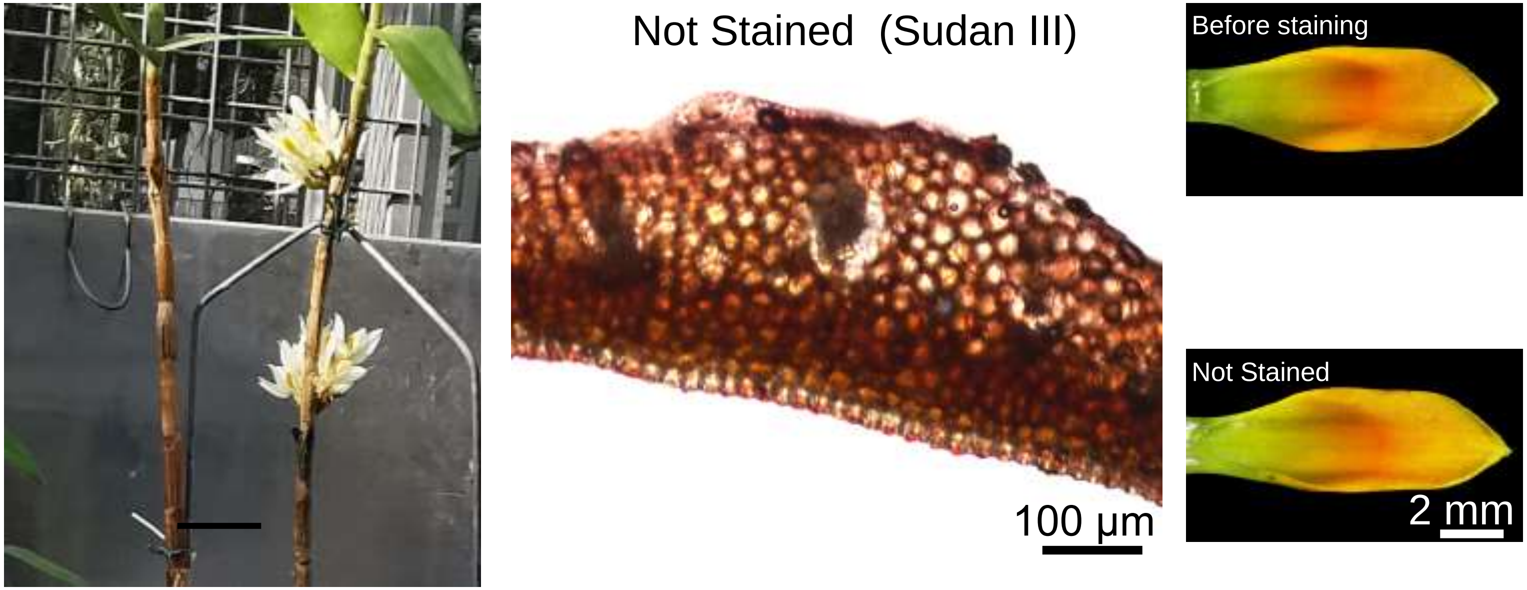

Dendrobium bracteosum; S-Q. Huang 2020122201

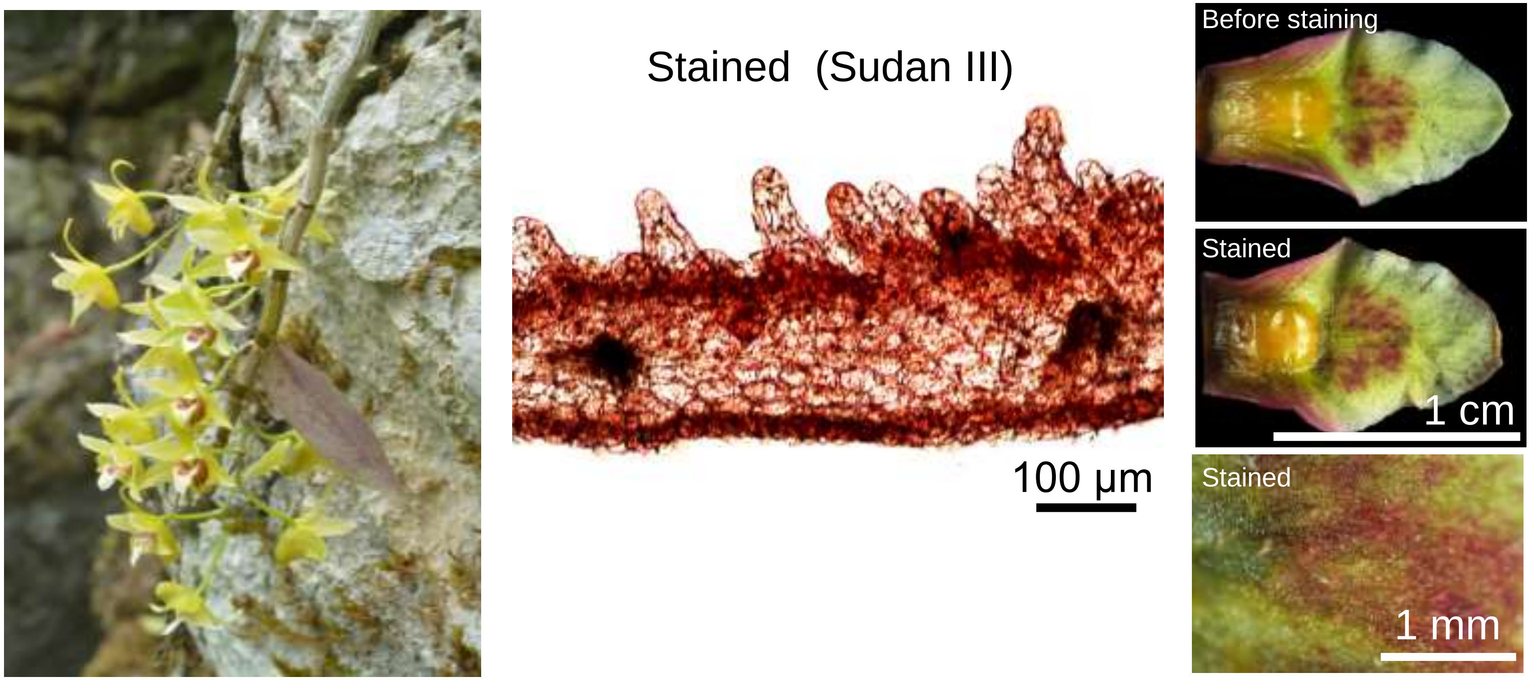

Dendrobium catenatum; S-Q. Huang 2020122202

Before staining   Not Stained (Sudan III)

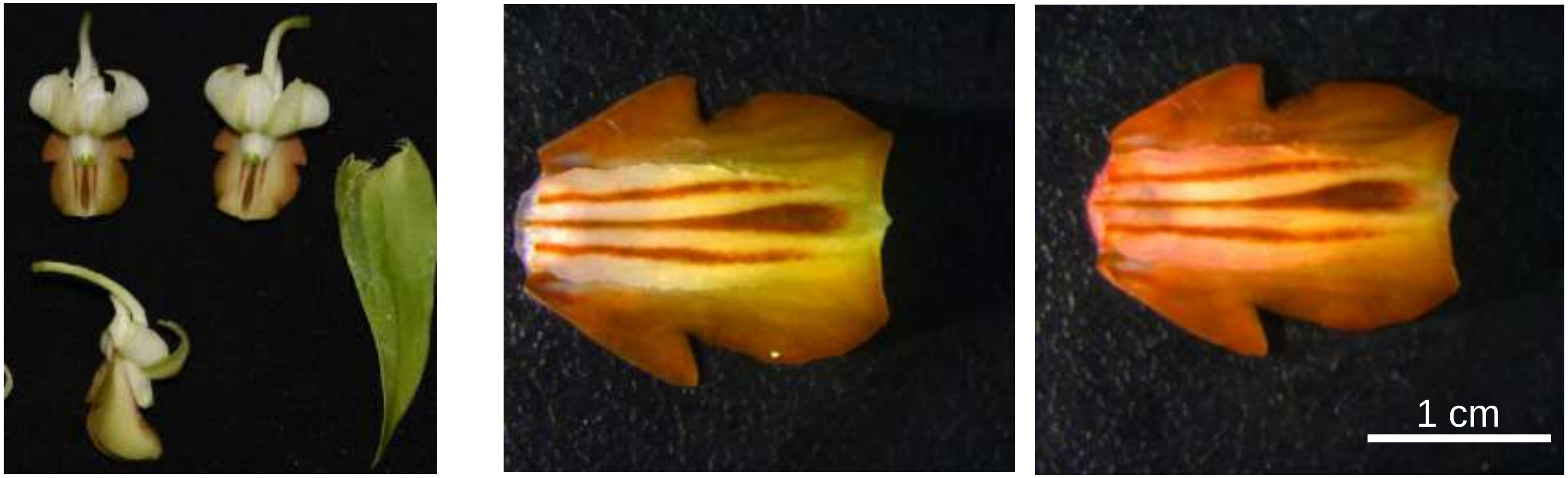

Dendrobium ellipsophyllum; M. Zhang 210402

Fixed in FAA   Stained (Sudan III)

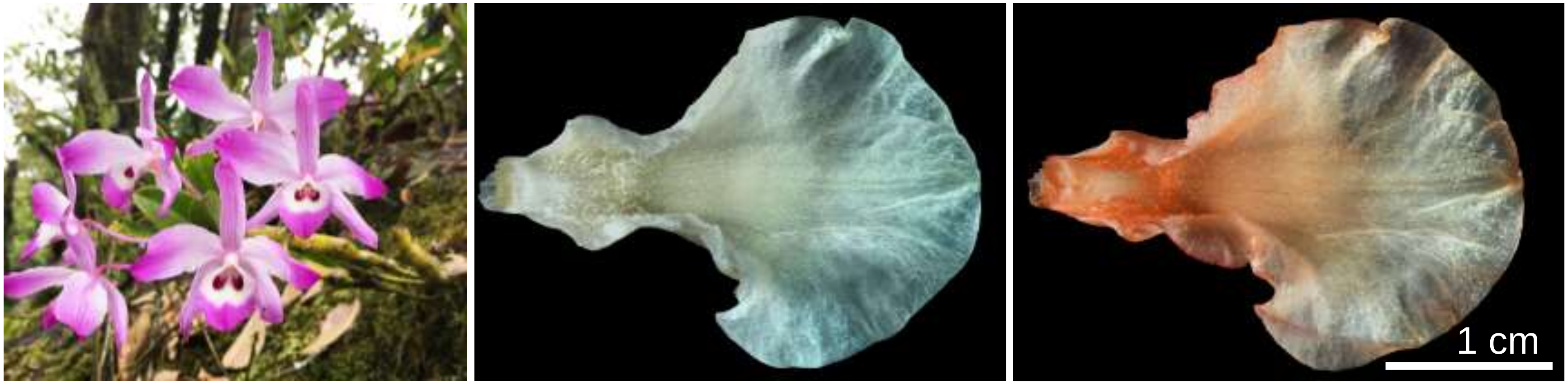

Dendrobium linawianum; Li-Bing Jia 20180509

Before staining Not Stained (Sudan III)

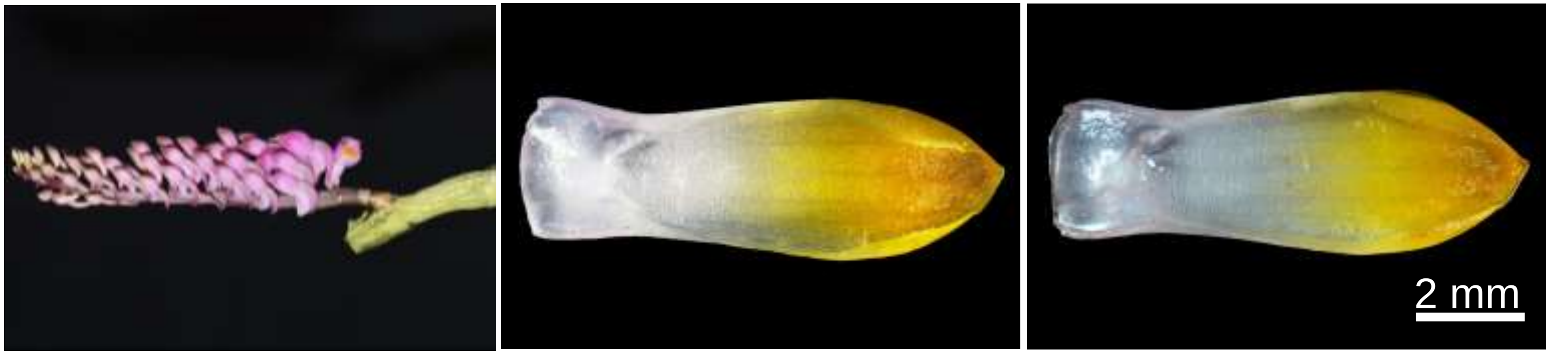

Dendrobium secundum; S-Q. Huang 2020122207

Stained (Sudan III)

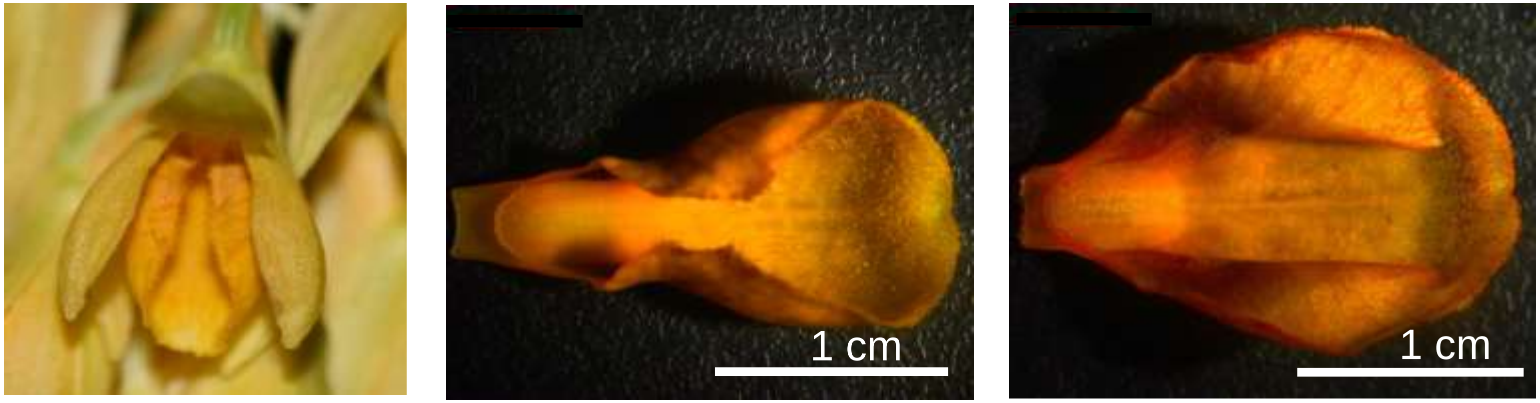

Dendrobium sulcatum; M. Zhang 210417

Before staining Stained (Sudan III)

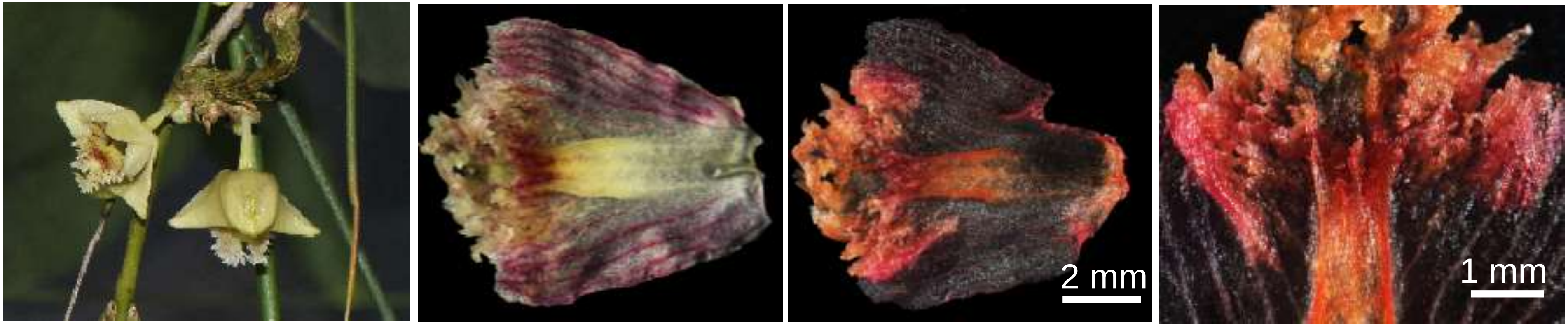

Dendrobium pseudotenellum; S-Q. Huang 2020122206

Before staining | Stained (Sudan III)

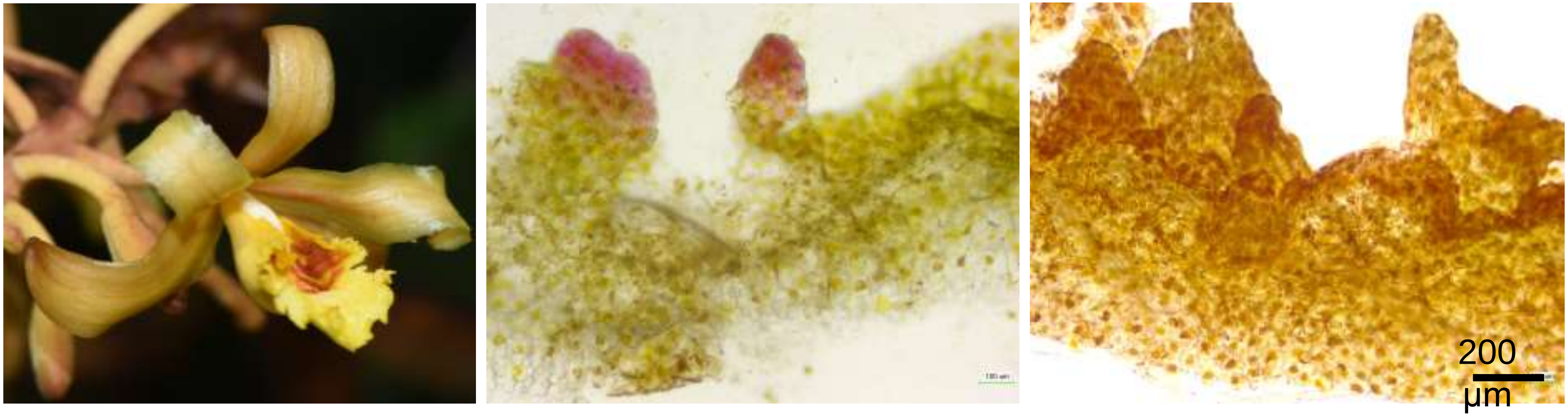

Galeola faberi; M. Zhang 230604

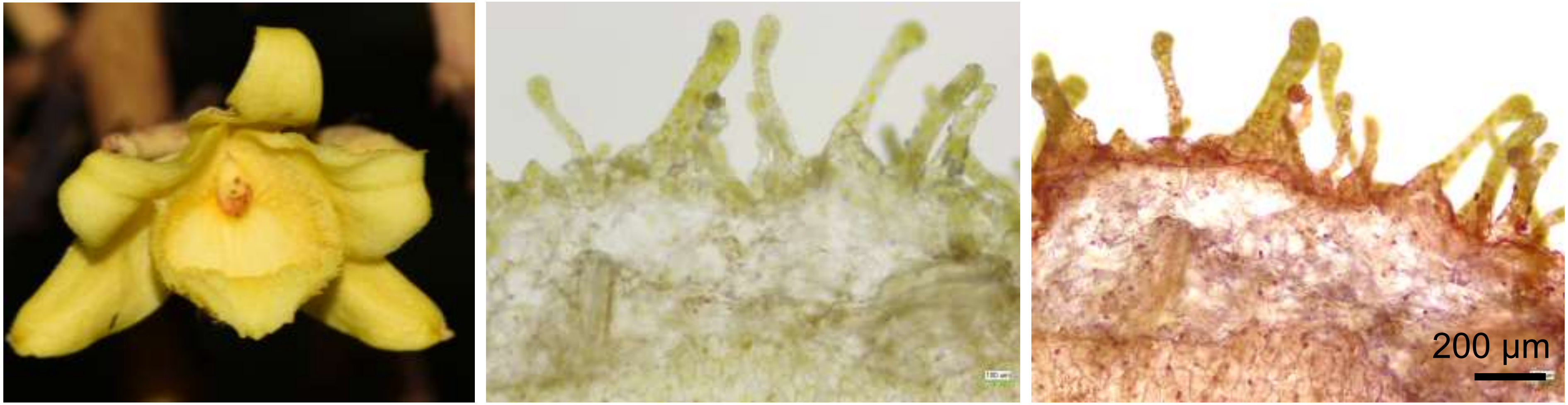

Galeola lindleyana; M. Zhang 230605
