# Supplementary material for: Multipartite oil-flower/oil-bee mutualisms involving male-bee-pollinated orchids in tropical Asia
Source: Natl Sci Rev. 2024 Feb 27;11(5):nwae072. doi: 10.1093/nsr/nwae072 (PMC11127695; doi:10.1093/nsr/nwae072)
Supplement: nwae072_Supplemental_Files [file nwae072_supplemental_files.zip › Supplementary Table Figure and Video.docx]

**Table S1**. Species of *Dendrobium*, *Galeola* (Orchidaceae), and Cucurbitaceae (bottom of table) observed in this study, with taxonomic authors, herbarium vouchers, geographic ranges, tests for the presence of nectar and/or oil, and observations of pollinators and pollinarium movement. The table includes previous observations on *Dendrobium* pollination in tropical Asia (Bartareau, 1993, 1994; Brodmann et al., 2009; Davies and Turner, 2004; Ho, 2008; Kjellsson et al., 1985; Pang et al., 2012; Salter and Calder, 1988). Note that the colour photo in Pang et al. (2012: Fig. 1C) of a bee labelled *Andrena parvula* does not show an *Andrena,* but instead a male *Ctenoplectra*, a genus distinguished by scopae with long simple hairs, an inner hind tibial spur expanded at its base (clearly seen in the paper’s Fig. 1C), and long hairs on the under-surfaces of the metasoma in the females (Vogel, 1990; Michener, 2007).

| **Species** | **Herbarium voucher**  **(CCNU)** | **Native range (Xiang et al., 2016)** | **Tested for nectar by observation and/or with pipettes** | **Tested for oil by Sudan III or IV staining** | **Pollinators** | **Pollinarium movement** |
| --- | --- | --- | --- | --- | --- | --- |
| *Dendrobium aduncum* Lindl. | Li-Bing Jia 20170616;  M. Zhang 220625 | China, Bhutan, India, Myanmar, Thailand, Vietnam | Without nectar (Jia & Huang 2022; this study) | Oil hairs present (this study) | Unknown | Not seen |
| *D. aphyllum* (Roxb.) C.E.C.Fisch. | S-Q. Huang 2020122203; M. Zhang 220404 | Hainan, China, Assam, Bangladesh, eastern Himalayas, India, Maldive | With nectar (this study) | Oil hairs present (this study) | *Ctenoplectra* spec*.* male seen in 2022 (this study) | Not seen |
| 1. *bracteosum* Rchb.f. | S-Q. Huang 2020122201 | Papua New Guinea | Unknown | No glandular hairs (this study) | Unknown | Unknown |
| *D. brymerianum* Rchb.f. | M. Zhang 220523 | SW China, Laos, Myanmar, Thailand, Vietnam | Without nectar (Jia & Huang 2022; this study) | Oil hairs present (this study) | *Ctenoplectra* *cornuta* females seen in 2021 (this study) | Pollinarium removal observed |
| *D. cariniferum* Rchb.f. | M. Zhang 220406 | SW China, NE India, Laos, Myanmar, N Thailand, Vietnam | Without nectar (Jia & Huang 2022; this study) | No oil (this study) | Unknown | Unknown |
| *D. catenatum* Lindl. | S-Q. Huang 2020122202 | SW & C China, Japan | Without nectar (Jia & Huang 2022) | Oil hairs present (this study) | Unknown | Unknown |
| *D. chrysanthum* Lindl. | M. Zhang 220733 | SW China, Bhutan, N India, Laos, Myanmar, Nepal, Thailand, Vietnam | With nectar (Jia & Huang 2022; this study) | Oil hairs present (this study) | *Ctenoplectra* *cornuta* males seen in 2021-2022 and females in 2021 (this study) | Pollinarium removal observed |
| *D.* *chryseum* Rolfe | M. Zhang 220522 | C Sichuan (Ebian, Emei Shan), Taiwan, NW to SE Yunnan [NE India, Myanmar]. | With nectar (this study) | Oil hairs present (this study) | *Ctenoplectra* *cornuta* males seen in 2016-2019, 2021-2022 and females in 2021-2022 (this study) | Pollinarium removal observed |
| *D. chrysotoxum* Lindl. | M. Zhang 220402 | SW China, NE India, Laos, Myanmar, Thailand, Vietnam | With nectar (Jia & Huang 2022; this study) | Oil hairs present (this study) | *Ctenoplectra* spec*.* male seen in 2022 (this study) | Not seen |
| *D. crepidatum* Lindl. & Paxton | M. Zhang 220407 | SW China, Bhutan, India, Laos, Myanmar, Nepal, Thailand, Vietnam | With nectar (Jia & Huang 2022) | Oil hairs present (this study) | Unknown | Unknown |
| *D. crumenatum* Sw. | M. Zhang 220416 | S China, Cambodia, India (Andaman Islands), Indonesia, Laos, Malaysia, Myanmar, Philippines, Sri Lanka, Thailand, Vietnam | Without nectar (Jia & Huang 2022; this study) | No glandular hairs (this study) | Unknown | Unknown |
| *D. crystallinum* Rchb.f. | M. Zhang 220417 | SW China, Cambodia, Laos, Myanmar, Thailand, Vietnam | Unknown | Oil hairs present (this study) | Unknown | Unknown |
| *D. denneanum* Kerr | M. Zhang 220524 | SW China, India, Laos, Myanmar, Nepal, Thailand, Vietnam | With nectar (Jia & Huang 2022; this study) | Oil hairs present (this study) | *Ctenoplectra cornuta* males seen in 2016-2018, 2021-2022 and females in 2021-2022 (this study) | Pollinarium removal observed |
| *D. densiflorum* Wall. | M. Zhang 220521 | Tropical China, Bhutan, NE India, Myanmar, Nepal, N Thailand | With nectar (Jia & Huang 2022; this study) | Oil hairs present (this study) | *Ctenoplectra* *cornuta* males seen in 2016-2018, 2021-2022 and females in 2022 (this study) | Pollinarium removal observed (video S2) |
| *D. devonianum* Paxton | M. Zhang 220627 | SW China, Bhutan, NE India, Myanmar, N Thailand, Vietnam | With nectar (Jia & Huang 2022; this study) | Oil hairs present (this study) | *Ctenoplectra* *cornuta* males seen in 2016 and 2021 (this study) | Pollinarium removal observed |
| *D. ellipsophyllum* T.Tang et F.T.Wang | M. Zhang 210402 | SW China, Cambodia, Laos, Myanmar, Thailand, Vietnam | Without nectar (Jia & Huang 2022) | No glandular hairs (this study) | Unknown | Unknown |
| *D. fimbriatum* Hook. | M. Zhang 220403 | SW China, Bhutan, India, Myanmar, Nepal, Thailand, Vietnam | With nectar (Jia & Huang 2022) | Oil hairs present (this study) | *Ctenoplectra* *cornuta* males seen in 2016 (this study) | Pollinarium removal observed |
| *D. gibsonii* Lindl. | M. Zhang 220630 | SW China, Bhutan, NE India, Myanmar, Nepal, N Thailand, Vietnam | With nectar (Jia & Huang 2022; this study) | Oil hairs present (this study) | *Ctenoplectra* *cornuta* males seen in 2016-2017 2021-2022, females in 2021 (this study) | Pollinarium removal observed |
| *D. hancockii* Rolfe | M. Zhang 220414 | China, N Vietnam | With nectar (Jia & Huang 2022) | Oil hairs present (this study) | Unknown | Unknown |
| *D. henryi* Schltr. | M. Zhang 220629 | SW China, Thailand, N Vietnam | Without nectar (this study) | Oil hairs present (this study) | Unknown | Pollinarium removal observed |
| *D. hercoglossum* Rchb.f. | M. Zhang 220628 | China, Laos, Malaysia, Thailand, Vietnam | Without nectar (Jia & Huang 2022; this study) | Oil hairs present (this study) | Unknown | Pollinarium removal observed |
| *D. infundibulum* Lindl. | N/A | SW China, SE India, Myanmar, Thailand, Laos; studied in Thailand (Kjellsson et al., 1985) | Without nectar (Kjellsson et al., 1985: visual inspection) | Unknown | *Bombus eximius*  (Kjellsson et al., 1985) | Pollinarium removal observed by Kjellsson et al. (1985) |
| *D. jiajiangense* Z.Y. Zhu, S.J. Zhu & H.B. Wang | N/A | Sichuan, China; studied in China (Pang et al., 2012) | Unknown (Pang et al., 2012; flowers not examined for nectar) | Glandular hairs on the labellum (Pang et al., 2012) | *Ctenoplectra* spec*.* (misidentified as *Andrena parvula* in Pang et al., 2012) | Pollinarium removal observed by Pang et al. (2012) |
| *D. kingianum* Bidw. | N/A | Australia | With nectar (Adams & Lawson 1987, 1988) | Unknown | *Apis mellifera*, ‘*Trigona*’ (Adams & Lawson 1987, 1988) | Pollinarium removal observed by Adams & Lawson (1987, 1988) |
| *D. linawianum* Rchb.f. | Li-Bing Jia 20180509 | Tropical China | Without nectar (Jia & Huang 2022) | Oil hairs present (this study) | Unknown | Unknown |
| *D. lindleyi* Steud. | M. Zhang 220834 | S China, Bhutan, India, Laos, Myanmar, Thailand, Vietnam | With nectar (this study) | Oil hairs present (this study) | Unknown | Unknown |
| *D. loddigesii* Rolfe | S-Q. Huang 2020122205; M. Zhang 220405 | China, Laos, N Vietnam | With nectar (Jia & Huang 2022) | Oil hairs present (this study) | *Ctenoplectra* *florisomnis* males or females unknown (He, 2008) | Pollinarium removal observed by He (2008) |
| *D. lohohense* Tang et F.T. Wang | M. Zhang 220520 | China | With nectar (Jia & Huang 2022; this study) | Oil hairs present (this study) | Unknown | Unknown |
| *D. longicornu* Lindl. | M. Zhang 220732 | SW China, Bhutan, NE India, Myanmar, Nepal, N Vietnam | Without nectar  (Jia & Huang 2022; this study) | No oil (this study) | Unknown | Pollinarium removal observed |
| *D.* *monophyllum* F.Muell. | N/A | E Australia; studied in Queensland (Bartareau, 1993) | Without nectar (Bartareau, 1993, visual inspection) | Unknown (flowers not tested) | *‘Trigona’* bees  (Bartareau, 1993)  Probably *Tetragonula carbonaria* (Smith) | Pollinarium removal observed by Bartareau, (1995) |
| *D. moschatum* (Buch.-Ham.) Sw. | M. Zhang 220418 | SW China, Bhutan, N India, Laos, Myanmar, Nepal, Thailand, Vietnam | With nectar (this study) | Oil hairs present (this study) | Unknown | Unknown |
| *D. nobile* Lindl. | M. Zhang 220410 | China, Bhutan, India, Laos, Myanmar, Nepal, N Thailand, Vietnam | Without nectar (Jia & Huang 2022; this study) | Oil hairs present (this study) | *Ctenoplectra* *cornuta* males seen in 2016, 2021-2022 and female in 2022 (this study) | Not seen |
| *D. pseudotenellum* Guillaumin | S-Q. Huang 2020122206;M. Zhang 211102 | SW China, Vietnam | Without nectar (this study) | Oil hairs present (this study) | Unknown | Unknown |
| *D. pulchellum* Lindl. | M. Zhang 220413 | Assam India, Bangladesh, Eastern Himalayas, Nepal, Myanmar, Thailand, Malaysia, Laos, SW China, Vietnam | Unknown | Oil hairs present (this study) | Unknown | Unknown |
| *D. scoriarum* W.W.Sm. | M. Zhang 220519 | SW China, Vietnam | Without nectar (Jia & Huang 2022; this study) | Oil hairs present (this study) | Unknown | Pollinarium removal observed |
| *D. secundum* (Blume) Lindl. | S-Q. Huang 2020122207 | SE Asia to Philippines, but not in China | Unknown | No oil (this study) | Unknown | Unknown |
| *D. signatum* Rchb.f. | M. Zhang 220408 | Burma, Thailand, Laos and Vietnam | Without nectar (Jia & Huang 2022) | Oil hairs present (this study) | Unknown | Unknown |
| *D. sinense* T. Tang et F.T. Wang | M. X. Ren 231031 | Tropical China; studied in Hainan (Brodmann et al., 2009) | Without nectar (this study) | No oil hairs present (this study) | *Vespa bicolor* hornets  (Brodmann et al., 2009) | Pollinarium removal observed by Brodmann et al. (2009) |
| *D. speciosum* Sm. | N/A | Tropical Australia, New Guinea; studied in Queensland (Salter and Calder, 1988; Adams, 1991) | Without nectar (Slater and Calder, 1988, visual inspection) | Unknown (flowers not tested) | *Homalictus brisbanensis* (Cockerell, 1918) (Salter and Calder, 1988; Adams, 1991) | Pollinarium removal observed by Salter and Calder (1988) |
| *D. stuposum* Lindl. | M. Zhang 220731 | SW China, Bhutan, NE India, Indonesia, Myanmar, Philippines, Thailand | With nectar (Jia & Huang 2022) | Oil hairs present (this study) | Unknown | Pollinarium removal observed |
| *D. sulcatum* Lindl. | M. Zhang 210417 | SW China, NE India, N Laos, Myanmar, N Thailand | With nectar (Jia & Huang 2022; this study) | Oil hairs present (this study) | Unknown | Unknown |
| *D. thyrsiflorum* B.S.Williams | M. Zhang 220401 | SW China, NE India, Lao, Myanmar, N Thailand, Vietnam | With nectar (Jia & Huang 2022; this study) | Oil hairs present (this study) | *Ctenoplectra* spec*.* males seen in 2022 (this study) | Not seen |
| *D. toressae* (Bailey) Dockr*.* | N/A | Tropical Australia; studied in Queensland (Bartareau, 1994) | Without nectar (Bartareau, 1994, visual inspection) | Unknown (flowers not tested) | *‘Trigona’* bees  (Bartareau, 1994)  Probably *Tetragonula carbonaria* (Smith) | Pollinarium removal observed by Bartareau (1994) |
| *D. transparens* Lindl. | M. Zhang 220415 | Western Himalayas, Bangladesh, Eastern Himalayas, Assam India, Nepal, Bhutan, Sikkim, Myanmar | Unknown | Oil hairs present (this study) | Unknown | Unknown |
| *D. unicum Seidenfaden* | N/A | Thailand | Without nectar (Davies and Turner, 2004) | Glandular hairs on the labellum (Davies and Turner, 2004) | Unknown | Unknown |
| *D. williamsonii* Day & Rchb.f. | M. Zhang 220626 | Chinese Himalayas, Assam India, Myanamar, Thailand, and Vietnam | Without nectar (this study) | No glandular hairs (this study) | Unknown | Unknown |
| *Galeola faberi* Rolfe | M. Zhang 230604 | C Guizhou, SW Sichuan, NW to SE Yunnan | Without nectar (this study) | Oil hairs present (this study) | Unknown | Unknown |
| *G. lindleyana* (Hook.f. & Thompson) Reichb.f. | M. Zhang 230605 | Anhui, W Guangdong, N Guangxi, Guizhou, Henan, Hunan, S Shaanxi, Sichuan, Taiwan, SE Xizang, W to SE Yunnan [Bhutan, India, Indonesia, Nepal] | Without nectar (this study) | Oil hairs present (this study) | *Ctenoplectra* *cornuta* males seen in 2023 (this study) | Pollinarium removal observed |
| **Species of Cucurbitaceae** | **Herbarium voucher**  **(CCNU)** | **Native range** | **Tested for nectar by observation and/or pipettes** | **Tested for oil by Sudan Ⅳ staining** | **Pollinators** | **Poll****en movement** |
| *Momordica subangulata* Blume | X.A. Wang 20220801 | Guangdong, Guangxi, Guizhou, Yunnan [Bangladesh, India, Indonesia, Laos, Malaysia, Myanmar, Thailand, Vietnam] | With nectar,  Vogel (1990),  this study | Oil hairs present, (Vogel, 1990),  this study | *Ctenoplectra* *cornuta* males and females seen in 2022 (this study) | Yes, this study (Fig. S3F) |
| *Thladiantha tomentosa* (A. M. Lu & Zhi Y. Zhang) W. Jiang & H. Wang | M. Zhang 230519 | Guanxi and Yunnan, south western China | With nectar (this study) | Oil hairs present,  this study | *Ctenoplectra* *cornuta* males and females in 2022 and 2023 (this study) | Yes, this study |
| *Thladiantha subglobosa* (Zhang et al., 2024) | X.A. Wang 2021070502 | Yunnan | With nectar (this study) | Oil hairs present (this study) | *Ctenoplectra* *cornuta* males and females in 2021 and 2023 (this study) | Yes, this study  (video S1) |

**References cited in Table S1**

Adams PB. Variation, multiple pollinators and breeding system in *Dendrobium speciosum* Sm.: A biological review. The Orchadian 1991, 10: 124-140.

Adams PB, Lawson SD. Pollination of *Dendrobium kingianum* Bidw. by the honey bee (*Apis mellifera*). The Orchadian 1987, 8: 250-251.

Adams PB, Lawson SD. Multiple bee pollinators of *Dendrobium kingianum* Bidw. in the natural habitat. The Orchadian 1988, 9: 103-107.

Bartareau T. Some observations on the pollination of *Dendrobium monophyllum* F. Muell. in north-east Queensland. The Orchadian 1993, 10: 446-450.

Bartareau T. The reproductive ecology of *Dendrobium toressae* (Bailey) Dockr., a geographically restricted species in North-east Queensland. The Orchadian 1994, 11:106-112.

Brodmann J, Twele R, Francke W, Luo YB, Song XQ, Ayasse M. Orchid mimics honey bee alarm pheromone in order to attract hornets for pollination. Curr. Biol. 2009, 19: 368-1372.

Davies KL, Turner MP. Pseudopollen in *Dendrobium unicum* Seidenf. (Orchidaceae): reward or deception? Annals of Botany 2004. 94: 129–132.

He PR. Study on orchids diversity in northern Hainan Island and pollination biology of *Dendrobium loddigesii*. A dissertation for Master degree in Guangxi Normal University. (In Chinese with English Abstract). 2008.

Kjellsson G, Rasmussen FN, Dupuy D. Pollination of *Dendrobium infundibulum*, *Cymbidium insigne* (Orchidaceae) and *Rhododendron lyi* (Ericaceae) by *Bombus eximius* (Apidae) in Thailand: a possible case of floral mimicry. J. Trop. Ecol. 1985, 1: 289-302.

Jia L-B, Huang S-Q. An examination of nectar production in 34 species of *Dendrobium* indicates that deceptive pollination in the orchids is not popular. J. Syst. Evol. 2022, 60: 1371–1377.

Pang SM, Pan KW, Wang YJ, Li W, Zhang L, Chen QB. Floral morphology and reproductive biology of *Dendrobium jiajiangese* (Orchidaceae) in Mt. Fotang Southwestern China. Flora 2012, 207: 469-474.

Schaefer H, Bartholomew B, Boufford DE. *Indofevillea jiroi* (Cucurbitaceae), a new floral oil-producing species from northeastern Myanmar. Harvard Papers Bot. 2012, 17:323–332.

Slater AT, Calder DM. The pollination biology of *Dendrobium speciosu*m Smith: a case of false advertising? Aust. J. Bot. 1988, 36: 145-158.

Vogel S. Ölblumen und Ölsammelnde Bienen. Dritte Folge. *Momordica*, *Thladiantha* und die Ctenoplectridae. Trop. u. Subtrop. Pflanzenwelt 1990,73: 1–186.

Zhang M, Qin Y, Renner SS, Schaefer H, Huang S-Q. *Thladiantha subglobosa* (Cucurbitaceae), a new floral-oil-offering species from southern China. Phytotaxa 2024, 637: 199-205.**Table S2 (a large pdf file, submitted separately).** Fresh, intact flowers of *Dendrobium* and *Galeola* stained with saturated ethanolic solutions of Sudan III or IV to detect the presence of accumulated lipids. For each species, a cross section of a labellum shows the glandular hairs before staining and after staining under a microscope or stereoscope. Where lipids could not be detected, this is indicated by ‘Not stained’. Voucher specimens are listed below the photos of each species and have been deposited in Herbarium of Central China Normal University (CCNU), see Table S1.

**Table S3**. Visits by male and female *Ctenoplectra cornuta* to flowers of *Dendrobium* or *Thladiantha*. (A) Visits to the *Dendrobium* species monitored at an orchid conservation centre in Malipo County or in experimental arrays (on the ground) 2-4 km away from the centre near flowering *Thladiantha* *subglobosa* (B) Visits by male and female *C. cornuta* to *D. chryseum* at the same two sites and (C) visits to male flowers of *T.* *subglobosa*.

| 1. *Ctenoplectra* *cornuta* visits to the *Dendrobium* species listed in Table S1. | | | | | | | | | | | | | | |  |
| --- | --- | --- | --- | --- | --- | --- | --- | --- | --- | --- | --- | --- | --- | --- | --- |
| Year | Hours of observation | Flower number | | Male bee | Female bee | | Males/ | | Females % | | Site | | | |  |
|  |  |  |  |  |  |  | Females | |  |  |  |  |  |  |  |
| 2016 | 163.4 | 6595 | | 40 | 0 | | - | | 0 | | Orchid centre | | | |  |
| 2017 | 57.0 | 4875 | | 4 | 0 | | - | | 0 | | Orchid centre | | | |  |
| 2021 | 17.3 | ca. 5000 | | 12 | 1 | | 12 | | 7.7 | | Orchid centre | | | |  |
| 2022 | 15.0 | ca. 4000 | | 5 | 2 | | 2.5 | | 28.6 | | Orchid centre | | | |  |
| 2023 | 30.0 | ca. 6000 | | 8 | 1 | | 8 | | 11.1 | | Orchid centre | | | |  |
| Sum | 282.7 | ca. 26470 | | 69 | 4 | | 17.3 | | 5.5 | | Orchid centre | | | |  |
| 2021 | 46.6 | 347 | | 97 | 13 | | 7.5 | | 11.8 | | With *Thladiantha* | | | |  |
| 2022 | 33.2 | 118 | | 48 | 20 | | 2.4 | | 29.4 | | With *Thladiantha* | | | |  |
| 2023 | 33.0 | 172 | | 51 | 3 | | 17 | | 5.6 | | With *Thladiantha* | | | |  |
| Sum | 112.8 | 637 | | 196 | 36 | | 5.4 | | 15.5 | | With *Thladiantha* | | | |  |
| Visit frequency increased (folds) | | | | 296 | | 937 | |  | |  | |  | | | |
| (B) *Ctenoplectra* *cornuta* visits to *Dendrobium chryseum.* | | | | | | | | | | | | |  |  |  |
| Year | Hours of observation | Flower number | | Male bee | Female bee | | Males/ | | Females % | | Site | | | |  |
|  |  |  |  |  |  |  | Females | |  |  |  |  |  |  |  |
| 2016 | 34.0 | 1072 | | 10 | 0 | | - | | 0 | | Orchid centre | | | |  |
| 2017 | 20.5 | 560 | | 1 | 0 | | - | | 0 | | Orchid centre | | | |  |
| 2018 | 29.5 | 893 | | 0 | 0 | | - | | - | | Orchid centre | | | |  |
| 2021 | 10.0 | 206 | | 3 | 0 | | - | | 0 | | Orchid centre | | | |  |
| 2022 | 9.0 | 184 | | 1 | 1 | | 1 | | 50.0 | | Orchid centre | | | |  |
| 2023 | 30.0 | 172 | | 2 | 0 | | - | | 0 | | Orchid centre | | | |  |
| Sum | 133.0 | 3087 | | 17 | 1 | | 17 | | 5.6 | | Orchid centre | | | |  |
| 2021 | 27.2 | 31 | | 15 | 5 | | 3 | | 25.0 | | With *Thladiantha* | | | |  |
| 2022 | 28.1 | 55 | | 47 | 18 | | 2.6 | | 27.7 | | With *Thladiantha* | | | |  |
| 2023 | 30.0 | 93 | | 7 | 2 | | 3.5 | | 22.2 | | With *Thladiantha* | | | |  |
| Sum | 85.3 | 179 | | 69 | 25 | | 2.8 | | 26.6 | | With *Thladiantha* | | | |  |
| Visit frequency increased (folds) | | | | 109 | | 672 | |  | |  | |  | | | |
| 1. *Ctenoplectra* *cornuta* visits to *Thladiantha* *subglobosa*. | | | | | | | | | | | | | | |  |
| Year | Hours of observation | | Flower number | Male bee | | Female bee | | Males/ | | Females % | | Site | | |  |
|  |  |  |  |  |  |  |  | Females | |  |  |  |  |  |  |
| 2021 | 41.6 | | 70 | 71 | | 16 | | 4.4 | | 18.4 | | With *Thladiantha* | | |  |
| 2022 | 26.2 | | 51 | 34 | | 55 | | 0.6 | | 61.8 | | With *Thladiantha* | | |  |
| 2023 | 28 | | 99 | 1 | | 29 | | 0.0 | | 96.7 | | With *Thladiantha* | | |  |
| Sum | 95.8 | | 220 | 106 | | 100 | | 1.1 | | 48.5 | | With *Thladiantha* | | |  |

**Table S4.** (A) Sizes (mean ± SE) of female (n = 3) and male (n = 3) *Ctenoplectra cornuta* bees compared with nonparametric Kruskal–Wallis tests. (B) Flower sizes of 17 *Dendrobium* species (mean ± SE, mm). Bold font indicates statistical significance.

(A)

| Traits (mm) | Male bee | Female bee | Test | p |
| --- | --- | --- | --- | --- |
| Antenna length | 2.44 ± 0.17 | 2.56 ± 0.05 | 0.429 | 0.5127 |
| Body length | 8.62 ± 0.30 | 8.92 ± 0.07 | 0.048 | 0.8273 |
| Head length | 1.29 ± 0.14 | 1.46 ± 0.23 | 1.190 | 0.2752 |
| Head width | 2.59 ± 0.09 | **2.90 ± 0.10** | **3.857** | **0.0495** |
| Thorax length | 3.44 ± 0.22 | 3.23 ± 0.06 | 0.429 | 0.5127 |
| Thorax width | 3.14 ± 0.11 | **3.56 ± 0.13** | **3.857** | **0.0495** |
| Abdomen length | 3.89 ± 0.18 | 4.22 ± 0.25 | 1.190 | 0.2752 |
| Abdomen width | 3.05 ± 0.07 | 3.13 ± 0.05 | 1.190 | 0.2752 |
| Abdomen hair length | 0.09 ± 0.003 | **0.56 ± 0.04** | **3.857** | **0.0495** |
| Tibia hair length | 0.28 ± 0.03 | **0.99 ± 0.10** | **3.857** | **0.0495** |
| Spur length | 0.78 ± 0.03 | **1.43 ± 0.08** | **3.857** | **0.0495** |
| Spur width | 0.11 ± 0.01 | **0.19 ± 0.02** | **3.857** | **0.0495** |
| Spur tooth length | 0.05 ± 0.003 | **0.09 ± 0.01** | **3.857** | **0.0495** |
| Spur tooth interval gap | 0.004 ± 0.0003 | **0.01 ± 0.001** | **3.857** | **0.0495** |

(B)

| Species | Flower length | Flower width | Labellum width | Entrance length | Entrance width | Spur length |
| --- | --- | --- | --- | --- | --- | --- |
| *D. aduncum* | 30.00 ± 0.45 | 30.16 ± 0.57 | 7.46 ± 0.05 |  |  | 11.48 ± 0.12 |
| *D. brymerianum* | 44.78 ± 0.58 | 27.86 ± 0.37 | 21.26 ± 0.43 | 4.37 ± 0.06 |  | 5.34 ± 0.09 |
| *D. catenatum* | 34.94 ± 0.55 | 25.69 ± 0.56 | 7.24 ± 0.07 | 4.30 ± 0.12 | 2.29 ± 0.05 | 9.54 ± 0.09 |
| *D. denneanum* | 48.72 ± 0.98 | 36.56 ± 0.87 | 21.93 ± 0.35 | 3.65 ± 0.11 | 2.78 ± 0.14 | 7.06 ± 0.10 |
| *D. densiflorum* | 34.78 ± 0.50 | 25.63 ± 0.38 | 16.90 ± 0.38 | 4.09 ± 0.06 | 2.19 ± 0.07 | 5.74 ± 0.09 |
| *D. devonianum* | 48.29 ± 0.74 | 39.98 ± 0.79 | 30.52 ± 0.60 | 4.46 ± 0.04 | 3.54 ± 0.06 | 6.12 ± 0.08 |
| *D. fimbriatum* | 51.63 ± 1.26 | 39.86 ± 0.69 | 27.73 ± 0.85 | 4.72 ± 0.06 | 3.57 ± 0.13 | 6.45 ± 0.09 |
| *D. gibsonii* | 35.33 ± 0.58 | 25.30 ± 0.45 | 19.23 ± 0.44 | 3.86 ± 0.05 | 2.67 ± 0.06 | 6.47 ± 0.10 |
| *D. hercoglossum* | 31.66 ± 0.54 | 28.36 ± 0.47 | 8.17 ± 0.08 | 4.25 ± 0.05 | 2.28 ± 0.08 | 3.94 ± 0.07 |
| *D. linawianum* | 65.29 ± 1.54 | 56.57 ± 1.34 | 19.57 ± 0.30 | 4.24 ± 0.06 | 3.29 ± 0.05 | 9.90 ± 0.11 |
| *D. loddigesii* | 38.95 ± 0.67 | 26.96 ± 0.61 | 20.03 ± 0.39 | 3.84 ± 0.08 | 3.22 ± 0.11 | 6.05 ± 0.08 |
| *D. lohohense* | 32.74 ± 0.53 | 25.78 ± 0.48 | 13.15 ± 0.18 | 3.78 ± 0.05 | 2.74 ± 0.06 | 6.21 ± 0.19 |
| *D. longicornu* | 23.91 ± 0.70 | 17.09 ± 0.43 | 14.64 ± 0.30 | 9.06 ± 0.14 | 6.23 ± 0.08 | 18.81 ± 0.40 |
| *D. nobie* | 77.95 ± 1.54 | 65.33 ± 1.87 | 26.81 ± 0.34 | 5.27 ± 0.08 | 4.17 ± 0.09 | 7.75 ± 0.14 |
| *D. scoriarum* | 26.71 ± 0.43 | 25.66 ± 0.37 | 7.70 ± 0.08 | 4.84 ± 0.06 | 2.09 ± 0.08 | 10.05 ± 0.09 |
| *D. stuposum* | 20.50 ± 0.30 | 13.25 ± 0.16 | 5.39 ± 0.10 | 4.15 ± 0.04 | 2.63 ± 0.04 | 5.88 ± 0.06 |
| *D. thyrsiflorum* | 38.10 ± 0.68 | 27.33 ± 0.62 | 16.33 ± 0.23 | 4.32 ± 0.06 | 2.87 ± 0.08 | 6.42 ± 0.12 |

**Table S5.** Nectar volume (mean ± SE, μL) and sugar concentration (%) per flower in *Dendrobium,* *Momordica subangulata* and *Thladiantha subglobosa*.

| Species | Nectar volume | Sugar concentration | Sampled flowers |
| --- | --- | --- | --- |
| *D. aduncum* | No | N/A | 15 |
| *D. brymerianum* | No | N/A | 20 |
| *D. chryseum* | 1.79 ± 0.07 | 24.7 ± 0.4 | 50 |
| *D. denneanum* | 1.20 ± 0.13 | 30.0 ± 1.5 | 15 |
| *D. densiflorum* | 1.39 ± 0.11 | 24.6 ± 1.2 | 25 |
| *D. devonianum* | 1.08 ± 0.2 | 24.5 ± 0.5 | 3 |
| *D. gibsonii* | 1.31 ± 0.17 | 31.5 ± 0.6 | 10 |
| *D. henryi* | No | N/A | 15 |
| *D. longicornu* | No | N/A | 15 |
| *D. nobile* | No | N/A | 20 |
| *D. scoriarum* | - | - | 5 |
| *Momordica subangulata* | 4.26 ± 0.28 | 44.8 ± 1.3 | 17 |
| *Thladiantha subglobosa* | 5.18 ± 0.51 | 36.0 ± 1.0 | 17 |

**Table S6.** Pollinarium removal and deposition in seven nectar-offering and six nectarless *Dendrobium* species. Extrapolating from the overall visitor spectrum, most pollinaria removals and depositions were due to male *Ctenoplectra* bees. Pollinaria removals per plant were significantly higher (Wald χ2 = 57.263, *P* < 0.001) in nectared species (Mean ± SE = 2.9 ± 0.2, n = 275 plants) than in nectarless species (0.4 ± 0.1, n = 147 plants). Pollinaria depositions per plant were also higher (Wald χ2 = 31.318, *P* < 0.001) in nectared species (1.1 ± 0.1, n = 350) than in nectarless species (0.1 ± 0.0, n = 130).

| Species | Nectar | No. plants | No.  flowers | Flowers per plant | Pollinaria removals per plant | Pollinaria depositions  per plant | No. plants | No. flowers |
| --- | --- | --- | --- | --- | --- | --- | --- | --- |
| *D. chrysanthum* | Yes | 54 | 553 | 10.2 ± 0.9 | 2.7 ± 0.6 | 0.6 ± 0.2 | 69 | 1682 |
| *D. chryseum* | Yes | 69 | 422 | 6.1 ± 0.3 | 0.8 ± 0.2 | 0.3 ± 0.1 | 67 | 564 |
| *D. denneanum* | Yes | 45 | 422 | 9.4 ± 0.3 | 3.1 ± 0.4 | 2.0 ± 0.3 | 75 | 734 |
| *D. densiflorum* | Yes | 8 | 152 | 19.0 ± 3.1 | 8.3 ± 2.4 | 2.3 ± 1.2 | 21 | 197 |
| *D. devonianum* | Yes | 6 | 30 | 5.0 ± 0.8 | 0.8 ± 0.3 | 0.3 ± 0.2 | 10 | 58 |
| *D. gibsonii* | Yes | 63 | 747 | 11.9 ± 0.6 | 5.9 ± 0.6 | 2.0 ± 0.4 | 86 | 1281 |
| *D. stuposum* | Yes | 30 | 67 | 2.2 ± 0.2 | 0.5 ± 0.1 | 0.1 ± 0.1 | 22 | 57 |
| *D. aduncum* | No | 5 | 45 | 9.0 ± 2.2 | 0 ± 0 | 0 ± 0 | 15 | 120 |
| *D. brymerianum* | No | 93 | 228 | 2.5 ± 0.1 | 0.3 ± 0.1 | 0 ± 0 | 16 | 38 |
| *D. henryi* | No | 28 | 52 | 1.9 ± 0.2 | 0.6 ± 0.2 | 0.3 ± 0.1 | 15 | 27 |
| *D. hercoglossum* | No | 10 | 26 | 2.6 ± 0.4 | 0.8 ± 0.3 | 0.2 ± 0.1 | 10 | 46 |
| *D. nobile* | No | 6 | 22 | 3.7 ± 0.9 | 0.2 ± 0.2 | 0 ± 0 | 48 | 396 |
| *D. scoriarum* | No | 5 | 6 | 1.2 ± 0.2 | 0.6 ± 0.2 | 0 ± 0 | 26 | 102 |


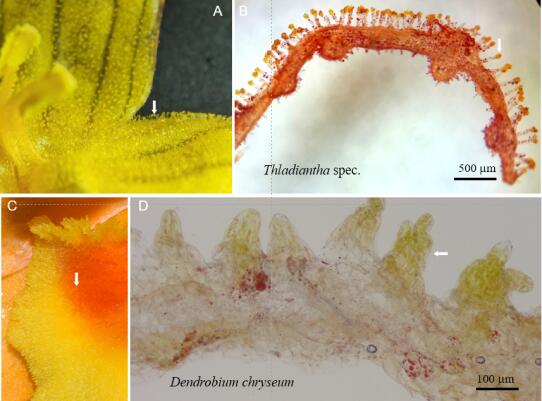


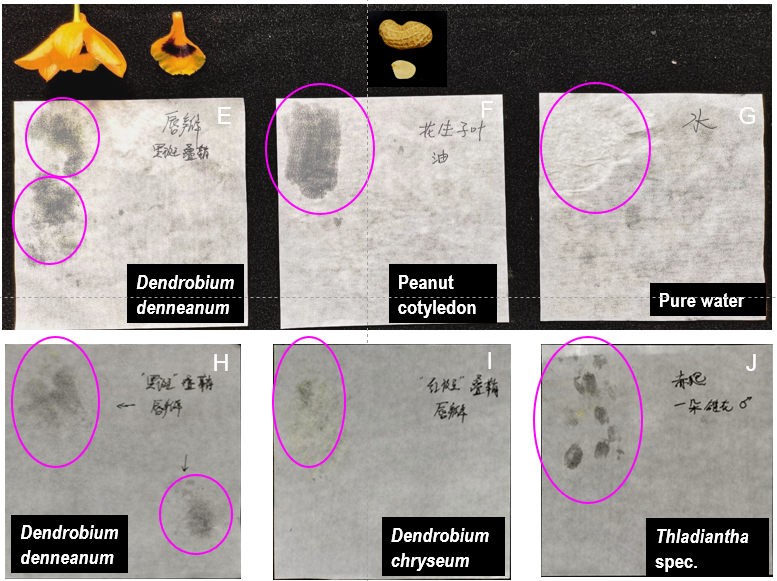


**Figure S1**. Comparison of floral oil secretion in *Dendrobium* *chryseum*, *D. denneanum*, and *Thladiantha subglobosa*. (A) The inner corolla of *Thladiantha* is covered by thousands of hairs (white arrow) and (B) in a hand-cut section stained with Sudan IV. (C) The orange-red patches on the labellum centre of *D.* *chryseum* and (D) a hand-cut section stained with Sudan IV under a microscope. (E) Oil stains (pink circles) left on weighing paper from a fresh labellum of *D. denneanum,* a piece of cotyledon of peanut (F), or 10 μL pure water (G) as control. Oil stains (pink circles) left on weighing paper by a fresh labellum of *D. denneanum* (H), *D.* *chryseum* (I), or five petals of one flower of *Thladiantha* (J).


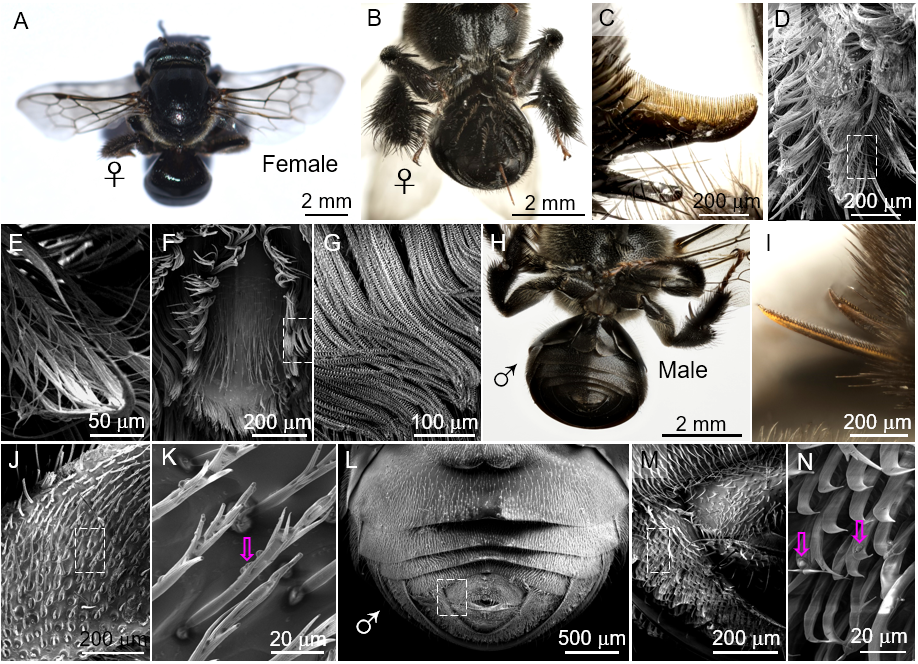
**Figure S2**. Morphological differences between female (A-G) and male (H-N) *Ctenoplectra cornuta*. (A) Dorsal view of a female bee. (B) Ventral metasoma of a female. (C) The oil-collecting tibial spur on the hindleg of a female. (D) Close-up of feather-shaped brushes on the tibial scopa on the hind leg. The rectangle marks an area shown further enlarged in (E). (F) Ventral metasoma of a female, and (G) further enlarged sternal view with oil mopping setae. (H) Ventral metasoma of a male. (I) Tibial spurs on the hindlegs of a male. (J) Close-up of the tibial scopa on the hind leg of a male, further enlarged in (K) the arrow marks an oil droplet. (L) Close-up of the ventral metasoma of a male. The rectangle marks an area shown further enlarged in (M). (N) Further enlarged area of (M) showing the oil-carrying hairs surrounding the abdomen tip with oil droplets still attached (pink arrows).


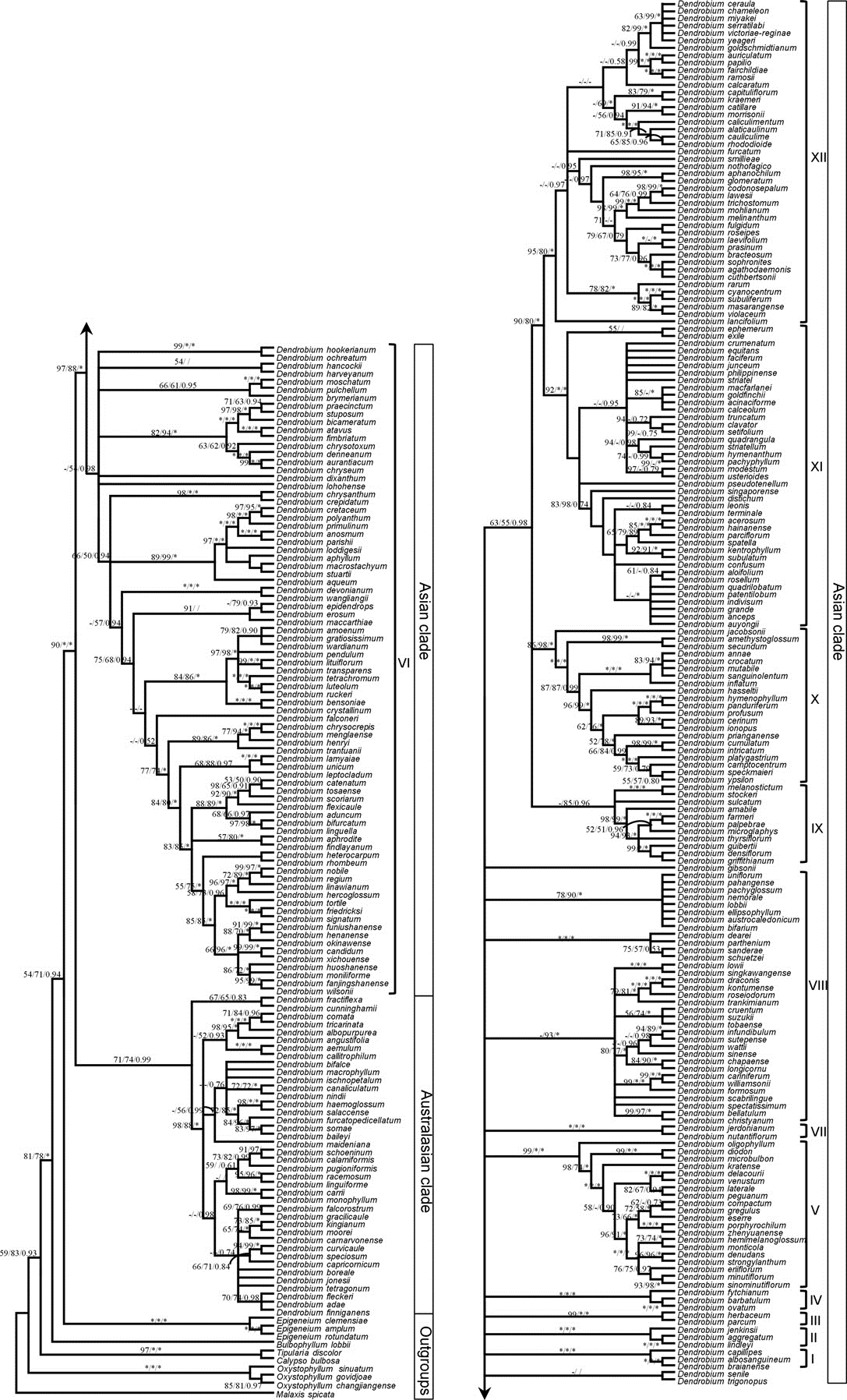


Nectar-offering

Nectarless

Oil-offering

Oilless

Unknown

**Figure S3**. Oil and nectar traits of 39 species studied here (Table S1) mapped onto a phylogeny of *Dendrobium* that strict consensus maximum parsimony tree based on five loci constructed by Xiang et al. (2016) includes 319 of the estimated 1200-1500 species. Numbers on branches are support values of maximum parsimony, maximum likelihood (ML) and Bayesian inference (BI) respectively, which asterisk (*) = 100%, dash (–) = < 50%, and blank = not appear in the ML or BI trees. Black triangle = oil-offering; open triangle = oilless; open square = unknown (presence of oil or nectar not tested); black circle = nectar-offering; open circle = nectarless. The *Dendrobium* species whose floral traits and pollinarium transfer were documented in this study (Table S1) represent different lineages in a molecular clock-dated phylogeny that species may date to the Pleistocene (Burke et al., 2008; Xiang et al., 2016). Although the statistical support for above phylogeny is low, shifts of oil-secreting hairs on the labellum may have evolved at least four times (blue circles). It thus appears that the interactions between male-*Ctenoplectra* bees and *Dendrobium* orchids could be relatively young.

**Video S1-S2**

**Video S1**. A female *Ctenoplectra cornuta* using its abdominal brushes to mop up oil from oil-offering glandular hairs on the inside corolla of *Thladiantha subglobosa*.

**Video S2.** A male *Ctenoplectra* *cornuta* collecting floral secretion from glandular hairs on the labellum of *Dendrobium densiflorum* after drinking nectar and leaving the flower with the pollinarium attached to its thorax.
